# Supplementary material for: Interventions to improve the quality of cataract services: protocol for a global scoping review
Source: BMJ Open. 2020 Aug 11;10(8):e036413. doi: 10.1136/bmjopen-2019-036413 (PMC7422650; doi:10.1136/bmjopen-2019-036413)
Supplement: Supplementary data [file bmjopen-2019-036413supp002.pdf]

Annex 2: Example search - MEDLINE

1. exp cataract/
2. Cataract Extraction/
3. cataract$.tw.
4. or/1-3
5. "Quality of Health Care"/
6. Quality Improvement/
7. Delivery of Health Care/
8. National Health Programs/
9. State Medicine/
10. Regional Health Planning/
11. Health Planning/
12. Health Plan Implementation/
13. Health Planning Guidelines/
14. Health Care Reform/
15. Health Resources/
16. Health Priorities/
17. Health Services Research/
18. "health services needs and demand"/
19. Needs Assessment/
20. State Health Plans/
21. Regional Health Planning/
22. Community Health Planning/
23. Hospital Planning/
24. Regional Medical Programs/
25. Health Maintenance Organizations/
26. Comprehensive Health Care/
27. Health Facility Planning/
28. Health Facility Administration/
29. Hospital Administration/
30. exp Hospitals, public/
31. exp Hospitals, private/
32. health system$.tw.

33. Models, Organizational/
34. Decision Making, Organizational/
35. Resource Allocation/
36. Efficiency, Organizational/
37. Organizational Innovation/
38. Delivery of Health Care, Integrated/
39. Interdisciplinary Communication/
40. Public Health/
41. Health Promotion/
42. Policy Making/
43. Program Development/
44. Program Evaluation/
45. Quality Control/
46. Quality Assurance, Health Care/
47. Benchmarking/
48. Capacity Building/
49. Health Services Accessibility/
50. Health Policy/
51. Surgical Procedures, Operative/
52. exp Surgical Equipment/
53. Health Care Rationing/
54. Medically Underserved Area/
55. Healthcare Disparities/
56. Health Status Disparities/
57. exp Attitude to Health/
58. "Patient Acceptance of Health Care"/
59. Health Education/
60. Public Opinion/
61. Health Behavior/
62. Social Behavior/
63. Superstitions/
64. exp Communication/
65. exp Culture/
66. Sex Factors/

67. Women's Rights/
68. Prejudice/
69. Vulnerable Populations/
70. Social Responsibility/
71. Social Welfare/
72. Urban Health Services/
73. Rural Health Services/
74. Rural Population/
75. (health adj20 (barrier$ or belie$ or inform$ or aware$ or knowledge or perceive$ or consequence$ or uptake or seek$ or underutili$ or fear$ or stigma$ or inequaliti$ or gender or logistic$ or distance$)).tw.
76. Patient Escort Service/
77. transport$.tw.
78. gender inequality.tw.
79. Mass Screening/
80. (referral adj3 (pathway$ or service$ or improve$)).tw.
81. (health worker$ or case finder$).tw.
82. or/5-81
83. Health Manpower/
84. Health Personnel/
85. Medical Staff, Hospital/
86. Nursing Staff, Hospital/
87. Personnel, Hospital/
88. Professional Competence/
89. Clinical Competence/
90. Medical Errors/
91. Professional Autonomy/
92. Leadership/
93. (leadership or motivat$).tw.
94. Motivation/
95. Organizational Innovation/
96. Personnel Selection/
97. Personnel Management/
98. Personnel Loyalty/

99. Job Satisfaction/
100. Staff Development/
101. "Attitude of Health Personnel"/
102. Personnel Turnover/
103. or/83-102
104. Clinical Governance/
105. Government Regulation/
106. Public Policy/
107. Public Health Practice/
108. Public Health Administration/
109. Health Plan Implementation/
110. Public-Private Sector Partnerships/
111. governance.tw.
112. or/104-111
113. (health management information system$ or HMIS).tw.
114. Management Information Systems/
115. Database Management Systems/
116. Computer Systems/
117. Point-of-Care Systems/
118. Hospital Information Systems/
119. Geographic Information Systems/
120. exp Medical Records Systems, Computerized/
121. Health Care Surveys/
122. Data Collection/
123. Data Interpretation, Statistical/
124. "Information Storage and Retrieval"/
125. Computer Literacy/
126. User-Computer Interface/
127. Attitude to Computers/
128. or/113-127
129. Delivery of Health Care, Integrated/
130. service delivery.tw.
131. decision making.tw.
132. (consensus adj3 (process$ or discuss)).tw.

133. stakeholder$.tw.
134. Quality Control/
135. Total Quality Management/
136. Quality Indicators, Health Care/
137. Quality Assurance, Health Care/
138. quality assurance.tw.
139. (quality adj2 improv$).tw.
140. total quality.tw.
141. continuous quality.tw.
142. quality management.tw.
143. (organisation$ adj3 cultur$).tw.
144. Disease Management/
145. Program Evaluation/
146. ((provider$ or program$) adj3 (monitor$ or evaluate$ or modif$ or practice)).tw.
147. (implement$ adj3 (improve$ or change$ or effort$ or issue$ or impede$ or glossary or tool$ or innovation$ or outcome$ or driv$ or examin$ or reexamin$ or scale$ or strateg$ or advis$ or expert$)).tw.
148. (needs adj3 assess$).tw.
149. ((education$ or learn$) adj5 (continu$ or material$ or meeting or collaborat$)).tw.
150. exp Medical audit/
151. (audit or feedback or compliance or adherence or training or innovation).ti.
152. (guideline$ adj3 (clinical or practice or implement$ or promot$)).tw.
153. exp Health Services Accessibility/
154. (outreach adj2 (service$ or visit$)).tw.
155. (intervention$ adj3 (no or usual or routine or target$ or tailor$ or mediat$)).tw.
156. usual care.tw.
157. exp Reminder Systems/
158. remind$.tw.
159. (improve$ adj3 (attend$ or visit$ or intervention$ or adhere$)).tw.
160. (increas$ adj3 (attend$ or visit$ or intervention$ or adhere$)).tw.
161. (appointment$ adj3 (miss$ or fail$ or remind$ or follow up)).tw.
162. Telephone/
163. telephone.tw.
164. Cell Phones/

165. Mobile Applications/
166. Remote Consultation/
167. (m-health or e-health or g-health or u-health).tw.
168. (phone$ adj1 (smart or cell)).tw.
169. (smartphone$ or cellphone$).tw.
170. (hand adj1 held device$).tw.
171. (mobile adj2 (health or healthcare or phone$ or device$ or monitor$ or comput$ or app or apps or application)).tw.
172. Internet/
173. Social Networking/
174. (email$ or text$ or message$).tw.
175. (letter or mail or mailed or print$ or brochure$ or newsletter$).tw.
176. Primary Health Care/
177. General Practitioners/ or Physicians, Family/ or Physicians, Primary Care/
178. Primary Prevention/
179. Preventive Health Services/
180. Community Health Services/
181. Community Health Nursing/
182. Health Services, Indigenous/
183. Rural Health Services/
184. Mobile Health Units/
185. (Ophthalmologist$ or Optometrist$ or Optician$ or Orthopist$ or Refractionists).tw.
186. ((Ophthalmic or eye) adj3 (surgeon$ or nurse$ or technician$ or officer$ or assistant$ or staff$)).tw.
187. Physician's Practice Patterns/
188. Professional Practice/
189. (professional adj3 (practice or develop$ or educat)).tw.
190. Education, Medical, Continuing/
191. exp nurses/
192. Specialties, Nursing/
193. Nurse's Role/
194. Education, Nursing, Continuing/
195. (nurse or nurses).tw.
196. Pharmacists/

197. pharmacist$.tw.
198. ((role or roles) adj3 expan$).tw.
199. (task$ adj3 shift$).tw.
200. exp Medical Records Systems, Computerized/
201. Management Information Systems/
202. Database Management Systems/
203. Computer Systems/
204. Point-of-Care Systems/
205. Hospital Information Systems/
206. ((health or healthcare) adj4 (record or management system$)).tw.
207. (decision adj5 support).ti.
208. Economics/
209. "costs and cost analysis"/
210. Cost allocation/
211. Cost-benefit analysis/
212. Cost control/
213. Cost savings/
214. Cost of illness/
215. Cost sharing/
216. "deductibles and coinsurance"/
217. Medical savings accounts/
218. Health care costs/
219. Direct service costs/
220. Drug costs/
221. Employer health costs/
222. Hospital costs/
223. Health expenditures/
224. Capital expenditures/
225. Value of life/
226. exp economics, hospital/
227. exp economics, medical/
228. Economics, nursing/
229. Economics, pharmaceutical/
230. exp "fees and charges"/

231. exp budgets/
232. (low adj cost).mp.
233. (high adj cost).mp.
234. (health?care adj cost$).mp.
235. (fiscal or funding or financial or finance).tw.
236. (cost adj estimate$).mp.
237. (cost adj variable).mp.
238. (unit adj cost$).mp.
239. (economic$ or pharmacoeconomic$ or price$ or pricing).tw.
240. Uncompensated Care/
241. Reimbursement Mechanisms/
242. Reimbursement, Incentive/
243. (insurance adj3 (health$ or scheme$)).tw.
244. (financial or economic or pay or payment or copayment or paid or fee or fees or monetary or money or cash or incentiv$ or disincentiv$).tw.
245. ((pay or paying or paid or cost$ or free or wait$ or qualit$) adj3 surg$).tw.
246. (will$ adj3 pay$).tw.
247. (waiting adj2 time).tw.
248. ((surgery or surgical) adj2 (experience or supervis$ or rate or rates or output or volume or uptake)).tw.
249. productivity.tw.
250. (patient adj3 (knowledge or satisfi$ or attitude$)).tw.
251. (percept$ adj3 quality).tw.
252. (follow up adj3 (appointment$ or poor or compliant or compliance)).tw.
253. exp Patient Acceptance of health Care/
254. exp Attitude to Health/
255. exp Health Behavior/
256. (barrier$ or obstacle$ or facilitat$ or enable$).tw.
257. (uptake or takeup or attend$ or accept$ or adhere$ or attitude$ or participat$ or facilitat$ or utilisat$ or utilizat$).tw.
258. (complie$ or comply or compliance$ or noncompliance$ or non compliance$).tw.
259. (encourag$ or discourage$ or reluctan$ or nonrespon$ or non respon$ or refuse$).tw.
260. (non-attend$ or non attend$ or dropout or drop out or apath$).tw.
261. Health Education/

262. exp Patient Education as Topic/
263. exp Health Promotion/
264. exp Counseling/
265. "Attitude of Health Personnel"/
266. (health adj2 (promotion$ or knowledge or belief$)).tw.
267. (educat$ adj2 (intervention$ or information or material or leaflet)).tw.
268. Socioeconomic Factors/
269. exp Poverty/
270. Social Class/
271. Educational Status/
272. ((school or education$) adj3 (status or level$ or attain$ or achieve$)).tw.
273. Employment/
274. Healthcare Disparities/
275. Health Status Disparities/
276. exp Medically Underserved Area/
277. Rural Population/
278. Urban Population/
279. exp Ethnic Groups/
280. Minority Groups/
281. Vulnerable Populations/
282. ((health$ or social$ or racial$ or ethnic$) adj5 (inequalit$ or inequit$ or disparit$ or equit$ or disadvantage$ or depriv$)).tw.
283. (disadvant$ or marginali$ or underserved or under served or impoverish$ or minorit$ or racial$ or ethnic$).tw.
284. (day adj3 (care or case$ or surger$)).tw.
285. (first eye adj1 cataract$).tw.
286. (second eye adj1 cataract$).tw.
287. (fellow eye adj1 cataract$).tw.
288. (simultaneous adj2 (phaco$ or phako$ or cataract$)).tw.
289. (bilateral adj2 (cataract$ surg$ or cataract$ extract$ or cataract$ remov$)).tw.
290. (sequential adj2 (cataract$ surg$ or cataract$ extract$ or cataract$ remov$)).tw.
291. Computer Simulation/
292. (virtual$ or simulat$).tw.
293. (residenc$ or resident$ or curriculum).tw.

294. or/129-293
295. 82 or 103 or 112 or 128 or 294
296. 4 and 295
297. epidemiologic studies/ or case-control studies/ or cohort studies/ or follow-up studies/ or longitudinal studies/ or prospective studies/ or controlled before-after studies/ or cross-sectional studies/ or historically controlled study/ or interrupted time series analysis/
298. epidemiologic methods/ or focus groups/ or interviews as topic/ or exp "surveys and questionnaires"/
299. epidemiologic research design/ or control groups/ or cross-over studies/ or double-blind method/ or meta-analysis as topic/ or network meta-analysis/ or random allocation/ or single-blind method/
300. epidemiologic methods/ or clinical trials as topic/ or feasibility studies/ or multicenter studies as topic/ or pilot projects/ or sampling studies/ or twin studies as topic/
301. randomized controlled trial/ or controlled clinical trials as topic/ or randomized controlled trials as topic/
302. comparative study/ or evaluation studies/ or meta-analysis/ or multicenter study/ or "systematic review"/ or validation studies/
303. Educational Measurement/
304. "Outcome and Process Assessment (Health Care)"/ or "Outcome Assessment (Health Care)"/

305. (cross adj1 section$).tw.
306. (cohort or intervention or prospective or comparative).tw.
307. (questionnaire$ or survey$).tw.
308. focus group$.tw.
309. (randomized or randomised or randomly).tw.
310. or/297-309
311. 296 and 310
312. (glaucoma$ or trabeculectom$ or angle closure or diabetic retinopath$ or keratoplast$ or keratopath$ or pseudoexfoliat$ or macula$ edema or macula$ oedema or retinal detachment$ or macula$ degeneration or scleral buckl$ or dry eye$ or uveitis or endothelial or endothelium or myopia or myopic or exotropia or amblyopia).ti.
313. (IOL$ or intraocular lens$ or trifocal or bifocal or multifocal or monofocal).ti.
314. (phacoemulsificat$ or capsulorhexis or wavefront or lensectomy or femtosecond or ECCE or SICS or MSICS or small incision or suture).ti.

315. (incidence or incident or prevalence).ti.
316. (dexamethasone or povidine or iodine or diclofenac or prednisolone or indomethacin or betaxolol or triamcinolone or nepafenac or corticosteroid$ or fluorouracil or bevacizumab or ranibizumab or radiation or ultrasound or intracameral or intravitreal or pseudophak$ or limbal or PMMA).ti.
317. optical coherence tomography.ti.
318. (genotyp$ or phenotyp$ or biomarker$ or genes or chromosome$ or mutation$).ti.
319. or/312-318
320. 311 not 319
321. exp case reports/
322. (case$ adj3 (report$ or stud$ or series)).tw.
323. 321 or 322
324. 320 not 323
325. limit 324 to (comment or editorial or letter or observational study)
326. 324 not 325
327. limit 326 to yr="1990 -Current"
