# Supplementary material for: Interventions to improve the quality of cataract services: protocol for a global scoping review
Source: BMJ Open. 2020 Aug 11;10(8):e036413. doi: 10.1136/bmjopen-2019-036413 (PMC7422650; doi:10.1136/bmjopen-2019-036413)
Supplement: Author's manuscript [file bmjopen-2019-036413.draft_revisions.pdf]

BMJ Open is committed to open peer review. As part of this commitment we make the peer review history of every article we publish publicly available.

When an article is published we post the peer reviewers' comments and the authors' responses online. We also post the versions of the paper that were used during peer review. These are the versions that the peer review comments apply to.

The versions of the paper that follow are the versions that were submitted during the peer review process. They are not the versions of record or the final published versions. They should not be cited or distributed as the published version of this manuscript.

BMJ Open is an open access journal and the full, final, typeset and author-corrected version of record of the manuscript is available on our site with no access controls, subscription charges or pay-per-view fees (http://bmjopen.bmj.com).

If you have any questions on BMJ Open's open peer review process please email info.bmjopen@bmj.com

BMJ Open

# Interventions to improve quality of cataract services: protocol for a scoping review



| | |
|---|---|
| Journal: | *BMJ Open* |
| Manuscript ID | bmjopen-2019-036413 |
| Article Type: | Protocol |
| Date Submitted by the Author: | 13-Dec-2019 |





| | |
|---|---|
| Complete List of Authors: | Yoshizaki, Miho; London School of Hygiene and Tropical Medicine International Centre for Eye Health<br>Ramke, Jacqueline; London School of Hygiene and Tropical Medicine International Centre for Eye Health; The University of Auckland, School of Optometry and Vision Science<br>Furtado, João; Universidade de São Paulo Faculdade de Medicina de Ribeirão Preto, Division of Ophthalmology<br>Burn, Helen; Stoke Mandeville Hospital<br>Gichuhi, Stephen; University of Nairobi, Department of Ophthalmology<br>Gordon, Iris; London School of Hygiene and Tropical Medicine International Centre for Eye Health<br>Aghaji, Ada; University of Nigeria, Department of Ophthalmology<br>Marques, Ana Patricia; London School of Hygiene and Tropical Medicine International Centre for Eye Health<br>Dean, William; London School of Hygiene and Tropical Medicine International Centre for Eye Health; University of Cape Town, Department of Ophthalmology<br>Congdon, Nathan; Queen's University Belfast, Centre for Public Health; Sun Yat-Sen University, Zhongshan Ophthalmic Center<br>Buchan, John; London School of Hygiene and Tropical Medicine International Centre for Eye Health<br>Burton, Matthew J; London School of Hygiene and Tropical Medicine International Centre for Eye Health; Moorfields Eye Hospital |





| | |
|---|---|
| Keywords: | Cataract and refractive surgery < OPHTHALMOLOGY, Quality in health care < HEALTH SERVICES ADMINISTRATION & MANAGEMENT, Health policy < HEALTH SERVICES ADMINISTRATION & MANAGEMENT |
| | |



SCHOLARONE™
Manuscripts

*I, the Submitting Author has the right to grant and does grant on behalf of all authors of the Work (as defined in the below author licence), an exclusive licence and/or a non-exclusive licence for contributions from authors who are: i) UK Crown employees; ii) where BMJ has agreed a CC-BY licence shall apply, and/or iii) in accordance with the terms applicable for US Federal Government officers or employees acting as part of their official duties; on a worldwide, perpetual, irrevocable, royalty-free basis to BMJ Publishing Group Ltd ("BMJ") its licensees and where the relevant Journal is co-owned by BMJ to the co-owners of the Journal, to publish the Work in this journal and any other BMJ products and to exploit all rights, as set out in our licence.*

*The Submitting Author accepts and understands that any supply made under these terms is made by BMJ to the Submitting Author unless you are acting as an employee on behalf of your employer or a postgraduate student of an affiliated institution which is paying any applicable article publishing charge ("APC") for Open Access articles. Where the Submitting Author wishes to make the Work available on an Open Access basis (and intends to pay the relevant APC), the terms of reuse of such Open Access shall be governed by a Creative Commons licence – details of these licences and which Creative Commons licence will apply to this Work are set out in our licence referred to above.*

*Other than as permitted in any relevant BMJ Author's Self Archiving Policies, I confirm this Work has not been accepted for publication elsewhere, is not being considered for publication elsewhere and does not duplicate material already published. I confirm all authors consent to publication of this Work and authorise the granting of this licence.*

For peer review only

# Interventions to improve quality of cataract services: protocol for a scoping review

Miho Yoshizaki[1], Jacqueline Ramke[1,2], João M. Furtado[3], Helen Burn[4], Stephen Gichuhi[5], Iris Gordon[1], Ada Aghaji[6], Ana Patricia Marques[1], William H Dean[1,7], Nathan Congdon[8,9], John Buchan[1], Matthew J Burton[1,10]

1. International Centre for Eye Health, London School of Hygiene & Tropical Medicine, London, United Kingdom
2. School of Optometry and Vision Science, University of Auckland, Auckland, New Zealand
3. Division of Ophthalmology, Ribeirão Preto Medical School, University of São Paulo, Ribeirão Preto, São Paulo, Brazil
4. Department of Ophthalmology, Stoke Mandeville Hospital, Aylesbury, United Kingdom
5. Department of Ophthalmology, University of Nairobi, Nairobi, Kenya
6. Department of Ophthalmology, College of Medicine, University of Nigeria, Nsukka, Nigeria
7. Department of Ophthalmology, University of Cape Town, Cape Town, South Africa
8. Centre for Public Health, Queens University Belfast, Belfast, UK
9. Zhongshan Ophthalmic Center, Sun Yat-sen University, Guangzhou, China
10. Moorfields Eye Hospital, London, United Kingdom

For peer review only



For peer review only - http://bmjopen.bmj.com/site/about/guidelines.xhtml

## ABSTRACT

### Introduction

Cataract is the leading cause of blindness globally, and a major cause of vision impairment. Cataract surgery is an efficacious intervention that usually restores vision. Although it is one of the most commonly conducted surgical interventions worldwide, good quality services (from being detected with operable cataract, to undergoing surgery and receiving post-operative care) are not universally available. Poor quality understandably reduces the willingness of people with operable cataract to undergo surgery. Therefore, it is critical to improve quality of care to subsequently reduce vision loss from cataract. This scoping review aims to summarise the nature and extent of the published literature on interventions to improve the quality of services for primary age-related cataract globally.

### Methods and analysis

We will search MEDLINE, Embase and Global Health for peer-reviewed manuscripts published since 1990, with no language, geographic or study design restrictions. To define quality, we have used the elements adopted by the World Health Organization—effectiveness, safety, people-centredness, timeliness, equity, integration and efficiency—to which we have added the element of planetary health. We will exclude studies focused on the technical aspects of the surgical procedure, and studies that only involve children (<18 years). Two reviewers will screen all titles/abstracts independently, followed by full-text review of potentially relevant articles. For included articles, data regarding publication characteristics, study details and quality related outcomes will be extracted by two reviewers independently. Results will be synthesised narratively and presented visually using a bubble diagram.

### Ethics and dissemination

Ethical approval is not required, as our review will only include published and publicly accessible information. We will publish our findings in an open-access peer-reviewed journal and develop an accessible summary of the results for website posting. A summary of the results will be included in the ongoing Lancet Global Health Commission on Global Eye Health.


**Registration details** https://osf.io/8gktz



For peer review only - http://bmjopen.bmj.com/site/about/guidelines.xhtml

## STRENGTHS AND LIMITATIONS OF THIS STUDY

- In many settings, quality of cataract services is only measured by post-operative visual acuity. One of the strengths of this review is the use of a broader concept of quality which includes the seven elements outlined in WHO's framework for healthcare quality, as well as the element of planetary health.
- Another strength is that we have broadened the scope of cataract services beyond the surgical intervention itself, to identify elements of service delivery that can impact on the broad range of quality elements.
- We have limited the review to interventions that occur in the non-operative period as we want to focus on interventions to improve detection, accessibility, uptake of surgery and overall outcome of the treatment across the service pathway. As such, we will exclude studies that report interventions involving intra-operative surgical and anaesthetic techniques, equipment and medication. This may be seen as a limitation by some, however this extensive literature is commonly synthesized in Cochrane and other reviews.
- A potential limitation is the paucity of published literature on interventions that address some elements of quality.

For peer review only



For peer review only - http://bmjopen.bmj.com/site/about/guidelines.xhtml

## INTRODUCTION

Cataract is the leading cause of blindness globally, and a major cause of moderate and severe vision impairment—an estimated 65 million people had vision loss from cataract in 2015.[1] Vision loss from cataract is unequally distributed throughout the world. For example, in 2015 among adults 50 years and above, the age standardized prevalence of cataract blindness ranged from 0.08% (80% uncertainty interval [UI] 0.03–0.19%) in high income countries of the Asia Pacific region to 2.35% (80% UI 0.72–5.04%) in West sub-Saharan Africa—almost a 30-fold difference.[1] Inequality (i.e. measurable differences between population subgroups) is also evident within countries, with a higher prevalence of cataract blindness among socially disadvantaged groups such as women, rural dwellers, and those who are not literate.[2]

Cataract surgery is an efficacious intervention that can restore vision[3-5] and alleviate poverty.[6] It is one of the most common surgical interventions in many high-income countries, and some middle-income countries.[7] However, good quality services are not universally available, particularly in low- and middle-income countries (LMICs).[8 9] Poor quality understandably reduces the willingness of people with operable cataract to undergo surgery.[10] Therefore it is critical to improve quality of care to subsequently reduce vision loss from cataract.

Quality of cataract services is most commonly measured using post-operative visual acuity. Measuring and monitoring outcomes is crucial in order to improve them[11] and tools are available to enable monitoring of post-operative visual acuity.[12]

Beyond using post-operative visual acuity to assess effectiveness, quality of cataract services includes many clinical and non-clinical dimensions.[13] For example:

- Timeliness: Cataract commonly occurs bilaterally. In many settings the current recommendation is to operate on one eye at a time and allow enough time for the operated eye to heal before operating on the second eye. However, delay in surgery for the second eye has been linked to increased risk of falls and road traffic accidents.[14]
- People-centredness: It may be common for patients to have to visit hospitals several times before the surgery for different pre-operative assessments, even though some of these could be done in one visit. Reducing the number of hospital visits to get surgery would improve quality from the patient perspective.
- Equity: There is no physiological reason why outcomes should be poorer in women compared to men, but women tend to have lower access and poorer post-operative vision outcomes



For peer review only - http://bmjopen.bmj.com/site/about/guidelines.xhtml

compared to men.[2 15] A further example of inequity is seen in the difference in effective cataract surgical coverage among Indigenous (51.6%, 95% confidence interval (CI) 42.4-60.7) and non-Indigenous Australians (88.5%, 95%CI 85.2-91.2).[16]

- Efficiency (Productivity): There is a link between the quantity of surgery a surgeon performs, and the quality of that surgery.[17] It has also been demonstrated that apparently cheaper service delivery options, such as outreach camps, can be less cost-effective compared to surgery delivered in static clinics due to worse outcomes.[18]

The aim of this review is to summarise the nature and extent of the published literature on interventions to improve the quality of cataract services globally. We chose to undertake a scoping review rather than an alternative evidence synthesis approach because we wished to identify and map the available evidence, which we anticipate will be heterogeneous.[19] We will take a broad perspective on quality outcomes and relevant interventions of interest, but will exclude studies focussed exclusively on the technical aspects of surgical techniques. For example, we will not include studies reporting effectiveness of phaco-emulsification or manual small incision surgery, as these are summarised in other reviews.[3-5 20]

**Definitions and framework development**

Cataract services includes the range of activities on the pathway from detecting people with operable cataract, to these people undergoing surgery and receiving post-operative care. As such, cataract services are both community and facility-based,[21] and—regardless of the setting—should involve a broad range of health care providers from the community level (e.g. village health workers as case-finders) through primary (e.g. optometrist) and secondary services (i.e. surgical team). In addition, consideration of all of the health system building blocks is relevant to strengthen cataract services.

Quality-of-care is one of the objectives embodied by the concept of Universal Health Coverage (UHC), together with equity in access and financial protection.[22] Our review will be guided by the definition of quality of care recently outlined by World Health Organisation (WHO):

> *Quality of care* is *'the degree to which health services for individuals and populations increase the likelihood of desired health outcomes and are consistent with current professional knowledge'.*[23]

WHO has adopted the framework of quality outlined by the Institute of Medicine.[24] This framework measures quality of healthcare across seven elements, as shown in Figure 1.



For peer review only - http://bmjopen.bmj.com/site/about/guidelines.xhtml

We have made one addition to the quality elements in WHO's framework—we believe that *planetary health* is an essential element of quality cataract surgery, so will also scope the literature on this. Planetary health is focused on sustainability, including the ability of the society to make choices while balancing the needs of future generations.[25]

To help guide the scope of our review, we mapped examples of outcome measures and interventions for cataract services against each of the eight elements of health care quality (Table 1). These outcomes and interventions were drawn from the literature,[26 27] as well as the knowledge and experience of the authorship group. For people-centredness, we drew on the outline of Integrated Person-Centred Health Services provided by WHO and adopted in the recent *World Report on Vision,* whereby services aim to provide coordinated care that addresses the full spectrum of eye conditions according to an individual's needs, and recognises people as participants and beneficiaries of this care.[28 29]

When mapping interventions, we categorised them using the WHO health systems "building blocks" i.e. we mapped them to the most relevant of Service delivery; Health workforce / Human Resources (HR), Health Information System (HIS); Access to essential consumables/non-consumables; Financing; and Leadership/governance. Recognising that this framework does not include community engagement and empowerment, we added *community* as an additional category against which interventions could be mapped.[30]



For peer review only - http://bmjopen.bmj.com/site/about/guidelines.xhtml

Table 1: Indicative outcomes and interventions to improve quality of cataract services*

| Quality elements | Description / notes | Example outcome measures | Example interventions |
|---|---|---|---|
| Effectiveness | WHO framework's defines this as adherence to evidence-based medicine.[23] | • Effective cataract surgical coverage[15]<br>• Pre and post-operative VA<br>• Contrast, glare, colour vision<br>• Years of sight-loss avoided | • ***Service delivery:*** day case vs. in-patient surgery; risk stratification of patients and matching with surgeon skills<br>• ***Equipment/consumables:*** pre-operative biometry correctly undertaken and interpreted; access to good quality range of IOL powers<br>• ***HIS***: recording and monitoring of outcomes - national data reporting system i.e. cataract surgery minimum dataset in UK and annual audit based on this data[31]; PRECOG[32]; BOOST[12]; national benchmarks for quality outcomes; post-operative spectacle supply |
| Safety | Patient harm is the 14th leading cause of global disease burden.[23] | • Intraoperative issues e.g. wrong lens insertion<br>• Post-operative issues e.g. endophthalmitis, cystoid macular oedema, retinal detachment, corneal oedema and decompensation incidents<br>• Refractive outcomes e.g. target spherical equivalent, prediction error, post-operative astigmatism | • ***Service delivery***: interventions to address surgical complications; protocols for emergency management of post-operative complications; post-operative care<br>• ***HR***: simulation training; continuing professional development for ophthalmologists<br>• ***HIS***: system to monitor individual surgeon performance;<br>• ***Governance:*** national benchmarks for quality outcomes in place (including refraction) quality assurance practice (i.e. WHO cataract check list, monitoring of outcomes)<br>• ***Equipment/consumables:*** IOL quality control, instrument sterilisation |
| People-centeredness | A good quality service should systematically incorporate needs and preferences of patients. | • Patient Reported Outcome Measures e.g. EQ-5D, HUI3, CatPROM5, Catquest-9SF, NEI VFQ-25<br>• Number of hospital attendances required | • ***Community:*** counselling about accessing surgery; informed consent process; social support (e.g. escort, family permission/support); dedicated eye health coordinators; pre-operative anxiety reduction strategies |
| Timeliness | Timely access to cataract surgery would improve patients experience and reduce the risk of complications. Early identification and appropriate referral is key to timely access. | • Severity of cataract at first presentation (including bilateral or unilateral)<br>• Time from diagnosis with operable cataract to completion of surgery<br>• Inter-operative time for patients with bilateral cataract | • ***Service delivery:*** re-design of pathways (diagnostics, referrals, treatment, follow-up) to be acceptable, affordable and sustainable; use of technology e.g. telemedicine; same-day bilateral surgery in low population density, low infection setting; strategies to reduce waiting list |
| Equity | Quality of care should not vary within a same setting according to patients' characteristics such | • Prevalence of cataract blindness and VI in sub-population (e.g. gender, | • ***Service delivery:*** outreach diagnostic protocols including consideration for false positives/negatives |



For peer review only - http://bmjopen.bmj.com/site/about/guidelines.xhtml

| | | | |
|---|---|---|---|
| | as age, gender, ethnicity, rural/urban and socio-economic status.<br><br>Equity can be considered in terms of equity of access to healthcare services or equity of health outcomes. | ethnic minority, indigeneity)<br>• Volume, distribution and effective coverage of surgery in sub-populations | • ***Equipment/consumables:*** reduced tax on imported items<br>• ***Community:*** financial support for patients who need it (i.e. subsidy for surgery, transport); patient information and education to raise awareness/anxiety management<br>• ***Financing:*** health insurance for cataract surgery[33] |
| Integration | Continuity of care and care coordination, including coordinating care for effectively managing comorbidities<br>Improve the care experience for people | • Referral pathways<br>• Multidisciplinary team training, accreditation and governance structure | • ***Service delivery:*** pathways (diagnostics, treatment, follow-up); support service; outreach and primary care screening diagnostic protocols / algorithms including consideration for false positives/negatives |
| Efficiency | Efficient use of resources, including productivity of surgeons, would contribute to quality improvement at population level.<br><br>Health service efficiency can be considered as allocative efficiency (optimal mix of inputs is being used to produce chosen outputs i.e. multi-disciplinary team, financial allocation) and technical efficiency (i.e. productivity of surgeons etc.) | • Productivity of surgeons (i.e. annual cataract operations per surgeon)<br>• Availability of manager/administrator<br>• Multi-disciplinary fixed/permanent team<br>• Financial management<br>• Cost-effectiveness analysis | • ***HR***: multidisciplinary team to support the surgeon - e.g. nurses seeing post-operative patients; task-shifting to non-ophthalmologist cataract surgeons; eye department manager; removing the need for specialist anaesthetist<br>• ***Financing***: financial sustainability of the providers; eye department autonomy over funds (budget and/or bank account); payment options that incentivise productivity and quality improvement (i.e. fee per service, bundled payment); modelling of cost recovery options that balance productivity, affordability and profit<br>• ***Equipment/consumables***: dedicated operating theatre |
| Planetary Health | Healthcare is a major consumer of energy and resources and produces considerable amounts of emissions and waste. In order to protect and improve the health and wellbeing of future generations, it needs to shift towards environmentally sustainable system. | | • ***Equipment/consumables***: reusable equipment, waste management<br>• ***HIS***: audit, lifecycle assessment<br>• ***Financing***: sustainable procurement |

*excluding surgical and aesthetical technical aspects, equipment and medication

For peer review only

## METHODS AND ANALYSIS

### Objectives / Scoping review questions

We aim to answer the following four questions:

1. What interventions to improve quality of cataract services have been described in the published literature?
2. Which element(s) of quality did the interventions address?
3. Where was the evidence generated (high- vs middle- vs low-income settings)?
4. What is the extent of the evidence for the effectiveness of these interventions?

### Protocol and registration

This protocol for this scoping review is reported according to the relevant sections of the PRISMA Extension for Scoping Reviews (PRISMA-ScR) guideline (Appendix 1).[34] The protocol is registered on the Open Science Framework (https://osf.io/8gktz).

### Eligibility criteria

This scoping review will include primary research studies and systematic reviews from any country that report a quality-relevant outcome for primary age-related cataract following an intervention related to quality of cataract services. Examples of relevant interventions are provided in Table 1, mapped against the eight quality elements of interest.

We will exclude studies assessing specific surgical techniques (e.g. phaco-emulsification versus manual small incision surgery, site of anaesthesia, size of incision) and/or specific products and medications used during surgery (e.g. monofocal versus multifocal intraocular lens, drug A versus drug B) as these are typically addressed in other systematic reviews.[3-5 20] Studies focussed exclusively on cataract services for children (aged under 18 years) will be excluded, as these services differ substantially from those for age-related cataract. We will also exclude studies reporting interventions to prevent cataract formation or progression. We will exclude studies published prior to 1990, as during the last 30 years there have been a large number of major developments in cataract surgery that would be expected to have changed the “landscape” substantially. Service delivery models prior to this time are quite different to those currently used. There will be no language limitations. Only studies where the full text is available will be included.

### Search

We will search MEDLINE, Embase and Global Health databases using search strategies developed by a Cochrane Eyes and Vision Information Specialist (IG). The search strategy for



For peer review only - http://bmjopen.bmj.com/site/about/guidelines.xhtml

MEDLINE is included in Appendix 2. We will examine reference lists of all included articles to identify further potentially relevant reports of studies. Field experts will be provided a list of the included studies and requested to identify further potentially relevant studies for consideration in the review.

**Selection of sources of evidence**

Covidence systematic review software will be used for screening (Veritas Health Innovation, Melbourne, Australia. Available at www.covidence.org). Each title and abstract will be screened independently by two reviewers (MY, JR, HB, AA, JB, JF, SG, WD) to exclude publications that clearly do not meet the inclusion criteria. Subsequently, the full text article will be retrieved for review if the citation seems potentially relevant and two reviewers will independently assess each article against the inclusion and exclusion criteria. Any discrepancies between the reviewers will be resolved by discussion, and a third reviewer will be consulted if necessary. A PRISMA flow diagram will be completed to summarise the study selection process.

**Data charting process**

A custom form will be developed in Excel for data charting. The form will be piloted on three studies and required amendments agreed by consensus. We anticipate a broad scope of included studies, so data charting will be an iterative process throughout the review and the data charting form will be amended as required. Each included study will be charted independently by two reviewers. Any discrepancies will be resolved by discussion, and a third reviewer will be consulted if necessary. We plan to contact study authors in the case of unclear information and will make up to three attempts by email.

**Data items**

The following data items will be collected during the data charting process:

1. Publication characteristics: title, year of publication, study design, country of origin, study setting;
2. Characteristics of intervention/study:
    a. Context (e.g. geographic area, target population and distribution, type of interventions (categorised by health system building block), target health practitioner, duration / frequency);
    b. Quality element(s) addressed by the intervention (as outlined in Table 1);
3. Outcome(s) of the intervention/study (examples outlined in Table 1).



For peer review only - http://bmjopen.bmj.com/site/about/guidelines.xhtml

### Synthesis of results

We recognise that the indication for surgery can vary across different settings due to the prevalence of vision loss from cataract, the capacity of services and the quality and safety standards in each setting. Accordingly, we will synthesize results by World Bank country income-level (high / upper-middle / lower-middle / low)[35] and (if possible) by GBD Super-Region (High income / Latin America & Caribbean / Sub-Saharan Africa / North Africa & Middle East / Southeast Asia, East Asia & Oceania / South Asia / Central Europe, Eastern Europe & Central Asia).[36]

We will summarise findings narratively and using descriptive statistical methods as appropriate. We will visualise the findings using a bubble diagram to show the extent of the evidence across each quality element (example shown in Figure 2). Where sufficient evidence is identified on a specific intervention, we will undertake a meta-analysis.

### Patient and Public Involvement Statement

As this is a scoping review, this study will be done without patient and public involvement.

## ETHICS AND DISSEMINATION

Ethical approval is not required, as our review will only include published and publicly accessible information.

We will publish our findings in an open-access, peer-reviewed journal and develop an accessible summary of the results for website posting and stakeholder meetings. A summary of the results will also be included in the ongoing Lancet Commission on Global Eye Health.[37]



For peer review only - http://bmjopen.bmj.com/site/about/guidelines.xhtml

## References


1. Flaxman S, Bourne R, Resnikoff S, et al. Global Causes of Distance Vision Loss: 1990-2015 and projections to 2020. *Lancet Glob Health* 2017;5(12):e1221-e34.
2. Ramke J, Zwi AB, Lee AC, et al. Inequality in cataract blindness and services: moving beyond unidimensional analyses of social position *Br J Ophthalmol* 2017;101(4):395-400.
3. De Silva S, Riaz Y, Evans J. Phacoemulsification with posterior chamber intraocular lens versus extracapsular cataract extraction (ECCE) with posterior chamber intraocular lens for age-related cataract. *Cochrane Database Syst Rev* 2014(1)
4. De Silva S, Riaz Y, Evans J. Phacoemulsification with posterior chamber intraocular lens versus extracapsular cataract extraction (ECCE) with posterior chamber intraocular lens for age-related cataract. *Cochrane Database Syst Rev* 2014(1)
5. Riaz Y, De Silva S, Evans J. Manual small incision cataract surgery (MSICS) with posterior chamber intraocular lens versus phacoemulsification with posterior chamber intraocular lens for age-related cataract. 2013(10)
6. Kuper H, Polack S, Mathenge W, et al. Does cataract surgery alleviate poverty? Evidence from a multi-centre intervention study conducted in Kenya, the Philippines and Bangladesh. 2010;5(11):e15431.
7. Eurostat. Surgical operations and procedures statistics. Available from: https://ec.europa.eu/eurostat/statistics-explained/index.php?title=Surgical_operations_and_procedures_statistics (accessed 15 November 2019).
8. Zhao J, Xu X, Ellwein LB, et al. Cataract surgical coverage and visual acuity outcomes in rural China in 2014 and comparisons with the 2006 China Nine-Province Survey. *Am J Ophthalmol* 2018;193:62-70.
9. Silva JC, Mújica OJ, Vega E, et al. A comparative assessment of avoidable blindness and visual impairment in seven Latin American countries: prevalence, coverage, and inequality. *Rev Panam Salud Publica* 2015;37(10):13-20.
10. Yin Q, Hu A, Liang Y, et al. A two-site, population-based study of barriers to cataract surgery in rural China. *Invest Ophthalmol Vis Sci* 2009;50(3):1069-75.
11. Yorston D, Gichuhi S, Wood M, et al. Does prospective monitoring improve cataract surgery outcomes in Africa? *Br J Ophthalmol* 2002;86(5):543-47.
12. Congdon N, Suburaman G-B, Ravilla T, et al. Transforming research results into useful tools for global health: BOOST. *Lancet Glob Health* 2016;4(2):e96.
13. Donabedian A. The quality of care: how can it be assessed? *JAMA* 1988;260(12):1743-48.
14. Meuleners LB, Fraser ML, Ng J, et al. The impact of first-and second-eye cataract surgery on injurious falls that require hospitalisation: a whole-population study. *Age Ageing* 2013;43(3):341-46.
15. Ramke J, Gilbert C, Lee ACL, et al. Effective cataract surgical coverage: an indicator for measuring quality-of-care in the context of Universal Health Coverage *PLoS One* 2017;12(3):e0172342.
16. Keel S, Xie J, Foreman J, et al. Population-based assessment of visual acuity outcomes following cataract surgery in Australia: the National Eye Health Survey. *Br J Ophthalmol* 2018;102(10):1419-24.
17. Bell CM, Hatch WV, Cernat G, et al. Surgeon volumes and selected patient outcomes in cataract surgery: a population-based analysis. *Ophthalmology* 2007;114(3):405-10.
18. Singh A, Garner P, Floyd K. Cost-effectiveness of public-funded options for cataract surgery in Mysore, India. *Lancet* 2000;355(9199):180-84.
19. Munn Z, Peters MD, Stern C, et al. Systematic review or scoping review? Guidance for authors when choosing between a systematic or scoping review approach. *BMC Med Res Methodol* 2018;18(1):143.




For peer review only - http://bmjopen.bmj.com/site/about/guidelines.xhtml

20. Riaz Y, Mehta J, Wormald R, et al. Surgical interventions for age-related cataract. *Cochrane Database Syst Rev* 2006(4)
21. World Health Organization. Strategies for the prevention of blindness in national programmes: a primary health care approach (2nd ed). Geneva: World Health Organization 1997.
22. World Health Organization. What is universal coverage? 2016 Accessed 1 August 2017]. Available from: http://www.who.int/health_financing/universal_coverage_definition/en/).
23. World Health Organization. Delivering quality health services: a global imperative for universal health coverage. Geneva: WHO, 2018.
24. Institute of Medicine Committee on Health Care in America. Crossing the quality chasm: A new health system for the 21st century. Washington DC: National Academies Press 2001.
25. Health TLP. The bigger picture of planetary health. *Lancet Planet Health* 2019;3(1):e1.
26. Buchan J, Dean W, Foster A, et al. What are the priorities for improving cataract surgical outcomes in Africa? Results of a Delphi exercise. *Int Ophthalmol* 2018;38(4):1409-14.
27. Lindfield R, Vishwanath K, Ngounou F, et al. The challenges in improving outcome of cataract surgery in low and middle income countries. *Indian J Ophthalmol* 2012;60(5):464.
28. World Health Organization. Framework on integrated, people-centred health services. Geneva: WHO, 2016.
29. World Health Organization. World report on vision. Geneva: WHO, 2019.
30. Sacks E, Morrow M, Story WT, et al. Beyond the building blocks: integrating community roles into health systems frameworks to achieve health for all. *BMJ Glob Health* 2019;3(Suppl 3):e001384.
31. Day A, Donachie P, Sparrow J, et al. The Royal College of Ophthalmologists' National Ophthalmology Database study of cataract surgery: report 1, visual outcomes and complications. 2015;29(552-560)
32. Congdon N, Yan X, Lansingh V, et al. Assessment of cataract surgical outcomes in settings where follow-up is poor: PRECOG, a multicentre observational study. *Lancet Glob Health* 2013;1(1):e37-e45.
33. Ackuaku-Dogbe E, Yawson A, Biritwum R. Cataract surgical uptake among older adults in Ghana. *Ghana Med J* 2015;49(2):84-89.
34. Tricco AC, Lillie E, Zarin W, et al. PRISMA extension for scoping reviews (PRISMA-ScR): checklist and explanation. *Ann Intern Med* 2018;169(7):467-73.
35. World Bank. Country and lending groups. Available from: https://datahelpdesk.worldbank.org/knowledgebase/articles/906519-world-bank-country-and-lending-groups (accessed 2 November 2019).
36. Institute for Health Metrics and Evaluation. Frequently asked questions - what countries are in each regions? Available from: https://datahelpdesk.worldbank.org/knowledgebase/articles/906519-world-bank-country-and-lending-groups (accessed 15 November 2019).
37. Burton MJ, Faal HB, Ramke J, et al. Announcing The Lancet Global Health Commission on Global Eye Health. *Lancet Glob Health* 2019;7(12):e1612-e13.




For peer review only - http://bmjopen.bmj.com/site/about/guidelines.xhtml

**Authors' Contributions:** JR and MJB conceived the idea for the review. MY and JR drafted and revised the protocol with suggestions from MJB, NC, JMF, SG, HB, AA, APM, WD and JB. IG constructed the search.

**Corresponding Author:** Miho Yoshizaki (miho.yoshizaki1@lshtm.ac.uk)

**Funding Statement:** MJB is supported by the Wellcome Trust (207472/Z/17/Z). JR is a Commonwealth Rutherford Fellow, funded by the UK government through the Commonwealth Scholarship Commission in the UK. The Lancet Global Health Commission on Global Eye Health is supported by The Queen Elizabeth Diamond Jubilee Trust, Moorfields Eye Charity [grant number GR001061], NIHR Moorfields Biomedical Research Centre, Wellcome Trust, Sightsavers, The Fred Hollows Foundation, The SEVA Foundation, British Council for the Prevention of Blindness and Christian Blind Mission.

**Acknowledgements**:

**Competing Interests:** None declared

**Number of Tables**: 1

**Number of Figures:** 2

**Data Sharing Statement:** Data generated from this review will be available upon reasonable request from Jacqueline.Ramke@lshtm.ac.uk

**Keywords:** cataract, cataract services, quality, effectiveness, safety, person-centredness, timeliness, equity,

**Word Count: 1,896**



For peer review only

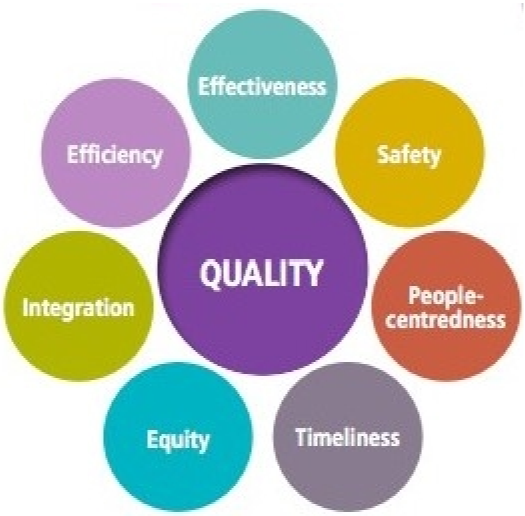


Elements of health care quality (reproduced from WHO, 2018)[24]

70x67mm (96 x 96 DPI)

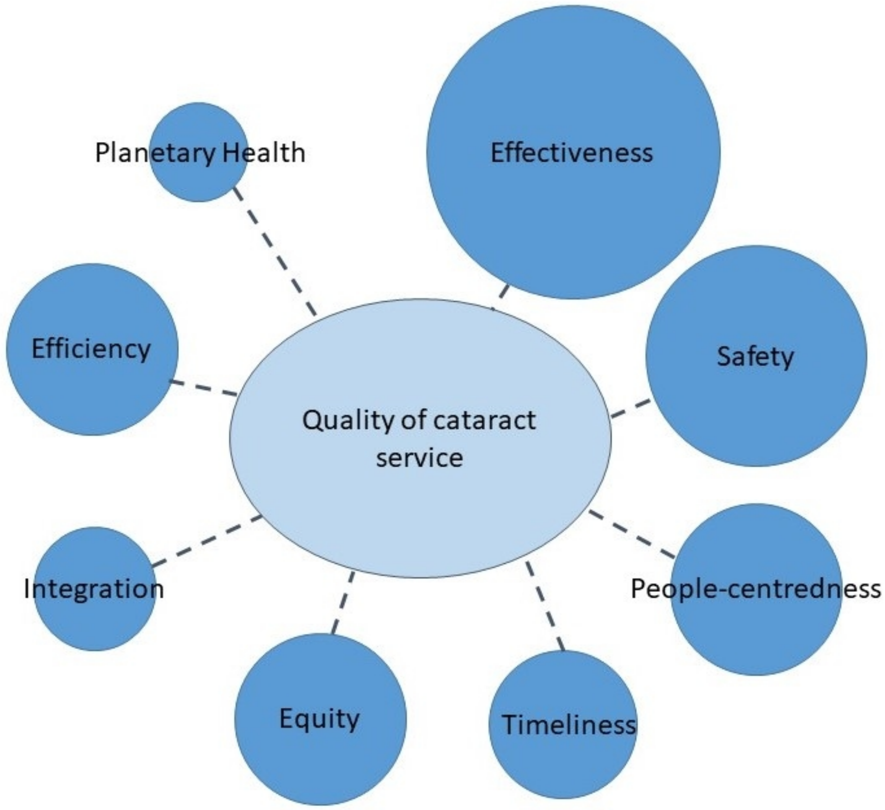


Example of bubble diagram mapping existing evidence of interventions improving cataract services

203x190mm (96 x 96 DPI)

## Annex 1: Preferred Reporting Items for Systematic reviews and Meta-Analyses extension for Scoping Reviews (PRISMA-ScR) Checklist

| SECTION | ITEM | PRISMA-ScR CHECKLIST ITEM | REPORTED ON PAGE # |
|---|---|---|---|
| **TITLE** | | | |
| Title | 1 | Identify the report as a scoping review. | 1 |
| **ABSTRACT** | | | |
| Structured summary | 2 | Provide a structured summary that includes (as applicable): background, objectives, eligibility criteria, sources of evidence, charting methods, results, and conclusions that relate to the review questions and objectives. | 2-3 |
| **INTRODUCTION** | | | |
| Rationale | 3 | Describe the rationale for the review in the context of what is already known. Explain why the review questions/objectives lend themselves to a scoping review approach. | 4-5 |
| Objectives | 4 | Provide an explicit statement of the questions and objectives being addressed with reference to their key elements (e.g., population or participants, concepts, and context) or other relevant key elements used to conceptualize the review questions and/or objectives. | 5-8 |
| **METHODS** | | | |
| Protocol and registration | 5 | Indicate whether a review protocol exists; state if and where it can be accessed (e.g., a Web address); and if available, provide registration information, including the registration number. | 9 |
| Eligibility criteria | 6 | Specify characteristics of the sources of evidence used as eligibility criteria (e.g., years considered, language, and publication status), and provide a rationale. | 9 |
| Information sources | 7 | Describe all information sources in the search (e.g., databases with dates of coverage and contact with authors to identify additional sources), as well as the date the most recent search was executed. | 9-10 |
| Search | 8 | Present the full electronic search strategy for at least 1 database, including any limits used, such that it could be repeated. | 9-10 |
| Selection of sources of evidence | 9 | State the process for selecting sources of evidence (i.e., screening and eligibility) included in the scoping review. | 10 |
| Data charting process | 10 | Describe the methods of charting data from the included sources of evidence (e.g., calibrated forms or forms that have been tested by the team before their use, and whether data charting was done independently or in duplicate) and any processes for obtaining and confirming data from investigators. | 10 |
| Data items | 11 | List and define all variables for which data were sought and any assumptions and simplifications made. | 10 |
| Critical appraisal of individual sources of evidence§ | 12 | If done, provide a rationale for conducting a critical appraisal of included sources of evidence; describe the methods used and how this information was used in any data synthesis (if appropriate). | N/A |
| Synthesis of results | 13 | Describe the methods of handling and summarizing the data that were charted. | 11 |

JBI = Joanna Briggs Institute; PRISMA-ScR = Preferred Reporting Items for Systematic reviews and Meta-Analyses extension for Scoping Reviews.

*From:* Tricco AC, Lillie E, Zarin W, O'Brien KK, Colquhoun H, Levac D, et al. PRISMA Extension for Scoping Reviews (PRISMA-ScR): Checklist and Explanation. Ann Intern Med. ;169:467–473. doi: 10.7326/M18-0850

For peer review only

**Annex 2: Example search - MEDLINE**

1. exp cataract/
2. Cataract Extraction/
3. cataract$.tw.
4. or/1-3
5. "Quality of Health Care"/
6. Quality Improvement/
7. Delivery of Health Care/
8. National Health Programs/
9. State Medicine/
10. Regional Health Planning/
11. Health Planning/
12. Health Plan Implementation/
13. Health Planning Guidelines/
14. Health Care Reform/
15. Health Resources/
16. Health Priorities/
17. Health Services Research/
18. "health services needs and demand"/
19. Needs Assessment/
20. State Health Plans/
21. Regional Health Planning/
22. Community Health Planning/
23. Hospital Planning/
24. Regional Medical Programs/
25. Health Maintenance Organizations/
26. Comprehensive Health Care/
27. Health Facility Planning/
28. Health Facility Administration/
29. Hospital Administration/
30. exp Hospitals, public/
31. exp Hospitals, private/
32. health system$.tw.

For peer review only

33. Models, Organizational/
34. Decision Making, Organizational/
35. Resource Allocation/
36. Efficiency, Organizational/
37. Organizational Innovation/
38. Delivery of Health Care, Integrated/
39. Interdisciplinary Communication/
40. Public Health/
41. Health Promotion/
42. Policy Making/
43. Program Development/
44. Program Evaluation/
45. Quality Control/
46. Quality Assurance, Health Care/
47. Benchmarking/
48. Capacity Building/
49. Health Services Accessibility/
50. Health Policy/
51. Surgical Procedures, Operative/
52. exp Surgical Equipment/
53. Health Care Rationing/
54. Medically Underserved Area/
55. Healthcare Disparities/
56. Health Status Disparities/
57. exp Attitude to Health/
58. "Patient Acceptance of Health Care"/
59. Health Education/
60. Public Opinion/
61. Health Behavior/
62. Social Behavior/
63. Superstitions/
64. exp Communication/
65. exp Culture/
66. Sex Factors/

For peer review only

67. Women's Rights/
68. Prejudice/
69. Vulnerable Populations/
70. Social Responsibility/
71. Social Welfare/
72. Urban Health Services/
73. Rural Health Services/
74. Rural Population/
75. (health adj20 (barrier$ or belie$ or inform$ or aware$ or knowledge or perceive$ or consequence$ or uptake or seek$ or underutili$ or fear$ or stigma$ or inequaliti$ or gender or logistic$ or distance$)).tw.
76. Patient Escort Service/
77. transport$.tw.
78. gender inequality.tw.
79. Mass Screening/
80. (referral adj3 (pathway$ or service$ or improve$)).tw.
81. (health worker$ or case finder$).tw.
82. or/5-81
83. Health Manpower/
84. Health Personnel/
85. Medical Staff, Hospital/
86. Nursing Staff, Hospital/
87. Personnel, Hospital/
88. Professional Competence/
89. Clinical Competence/
90. Medical Errors/
91. Professional Autonomy/
92. Leadership/
93. (leadership or motivat$).tw.
94. Motivation/
95. Organizational Innovation/
96. Personnel Selection/
97. Personnel Management/
98. Personnel Loyalty/

For peer review only

99. Job Satisfaction/
100. Staff Development/
101. "Attitude of Health Personnel"/
102. Personnel Turnover/
103. or/83-102
104. Clinical Governance/
105. Government Regulation/
106. Public Policy/
107. Public Health Practice/
108. Public Health Administration/
109. Health Plan Implementation/
110. Public-Private Sector Partnerships/
111. governance.tw.
112. or/104-111
113. (health management information system$ or HMIS).tw.
114. Management Information Systems/
115. Database Management Systems/
116. Computer Systems/
117. Point-of-Care Systems/
118. Hospital Information Systems/
119. Geographic Information Systems/
120. exp Medical Records Systems, Computerized/
121. Health Care Surveys/
122. Data Collection/
123. Data Interpretation, Statistical/
124. "Information Storage and Retrieval"/
125. Computer Literacy/
126. User-Computer Interface/
127. Attitude to Computers/
128. or/113-127
129. Delivery of Health Care, Integrated/
130. service delivery.tw.
131. decision making.tw.
132. (consensus adj3 (process$ or discuss)).tw.

For peer review only

133. stakeholder$.tw.
134. Quality Control/
135. Total Quality Management/
136. Quality Indicators, Health Care/
137. Quality Assurance, Health Care/
138. quality assurance.tw.
139. (quality adj2 improv$).tw.
140. total quality.tw.
141. continuous quality.tw.
142. quality management.tw.
143. (organisation$ adj3 cultur$).tw.
144. Disease Management/
145. Program Evaluation/
146. ((provider$ or program$) adj3 (monitor$ or evaluate$ or modif$ or practice)).tw.
147. (implement$ adj3 (improve$ or change$ or effort$ or issue$ or impede$ or glossary or tool$ or innovation$ or outcome$ or driv$ or examin$ or reexamin$ or scale$ or strateg$ or advis$ or expert$)).tw.
148. (needs adj3 assess$).tw.
149. ((education$ or learn$) adj5 (continu$ or material$ or meeting or collaborat$)).tw.
150. exp Medical audit/
151. (audit or feedback or compliance or adherence or training or innovation).ti.
152. (guideline$ adj3 (clinical or practice or implement$ or promot$)).tw.
153. exp Health Services Accessibility/
154. (outreach adj2 (service$ or visit$)).tw.
155. (intervention$ adj3 (no or usual or routine or target$ or tailor$ or mediat$)).tw.
156. usual care.tw.
157. exp Reminder Systems/
158. remind$.tw.
159. (improve$ adj3 (attend$ or visit$ or intervention$ or adhere$)).tw.
160. (increas$ adj3 (attend$ or visit$ or intervention$ or adhere$)).tw.
161. (appointment$ adj3 (miss$ or fail$ or remind$ or follow up)).tw.
162. Telephone/
163. telephone.tw.
164. Cell Phones/

For peer review only

165. Mobile Applications/
166. Remote Consultation/
167. (m-health or e-health or g-health or u-health).tw.
168. (phone$ adj1 (smart or cell)).tw.
169. (smartphone$ or cellphone$).tw.
170. (hand adj1 held device$).tw.
171. (mobile adj2 (health or healthcare or phone$ or device$ or monitor$ or comput$ or app or apps or application)).tw.
172. Internet/
173. Social Networking/
174. (email$ or text$ or message$).tw.
175. (letter or mail or mailed or print$ or brochure$ or newsletter$).tw.
176. Primary Health Care/
177. General Practitioners/ or Physicians, Family/ or Physicians, Primary Care/
178. Primary Prevention/
179. Preventive Health Services/
180. Community Health Services/
181. Community Health Nursing/
182. Health Services, Indigenous/
183. Rural Health Services/
184. Mobile Health Units/
185. (Ophthalmologist$ or Optometrist$ or Optician$ or Orthopist$ or Refractionists).tw.
186. ((Ophthalmic or eye) adj3 (surgeon$ or nurse$ or technician$ or officer$ or assistant$ or staff$)).tw.
187. Physician's Practice Patterns/
188. Professional Practice/
189. (professional adj3 (practice or develop$ or educat)).tw.
190. Education, Medical, Continuing/
191. exp nurses/
192. Specialties, Nursing/
193. Nurse's Role/
194. Education, Nursing, Continuing/
195. (nurse or nurses).tw.
196. Pharmacists/

For peer review only

197. pharmacist$.tw.
198. ((role or roles) adj3 expan$).tw.
199. (task$ adj3 shift$).tw.
200. exp Medical Records Systems, Computerized/
201. Management Information Systems/
202. Database Management Systems/
203. Computer Systems/
204. Point-of-Care Systems/
205. Hospital Information Systems/
206. ((health or healthcare) adj4 (record or management system$)).tw.
207. (decision adj5 support).ti.
208. Economics/
209. "costs and cost analysis"/
210. Cost allocation/
211. Cost-benefit analysis/
212. Cost control/
213. Cost savings/
214. Cost of illness/
215. Cost sharing/
216. "deductibles and coinsurance"/
217. Medical savings accounts/
218. Health care costs/
219. Direct service costs/
220. Drug costs/
221. Employer health costs/
222. Hospital costs/
223. Health expenditures/
224. Capital expenditures/
225. Value of life/
226. exp economics, hospital/
227. exp economics, medical/
228. Economics, nursing/
229. Economics, pharmaceutical/
230. exp "fees and charges"/

For peer review only

231. exp budgets/
232. (low adj cost).mp.
233. (high adj cost).mp.
234. (health?care adj cost$).mp.
235. (fiscal or funding or financial or finance).tw.
236. (cost adj estimate$).mp.
237. (cost adj variable).mp.
238. (unit adj cost$).mp.
239. (economic$ or pharmacoeconomic$ or price$ or pricing).tw.
240. Uncompensated Care/
241. Reimbursement Mechanisms/
242. Reimbursement, Incentive/
243. (insurance adj3 (health$ or scheme$)).tw.
244. (financial or economic or pay or payment or copayment or paid or fee or fees or monetary or money or cash or incentiv$ or disincentiv$).tw.
245. ((pay or paying or paid or cost$ or free or wait$ or qualit$) adj3 surg$).tw.
246. (will$ adj3 pay$).tw.
247. (waiting adj2 time).tw.
248. ((surgery or surgical) adj2 (experience or supervis$ or rate or rates or output or volume or uptake)).tw.
249. productivity.tw.
250. (patient adj3 (knowledge or satisfi$ or attitude$)).tw.
251. (percept$ adj3 quality).tw.
252. (follow up adj3 (appointment$ or poor or compliant or compliance)).tw.
253. exp Patient Acceptance of health Care/
254. exp Attitude to Health/
255. exp Health Behavior/
256. (barrier$ or obstacle$ or facilitat$ or enable$).tw.
257. (uptake or takeup or attend$ or accept$ or adhere$ or attitude$ or participat$ or facilitat$ or utilisat$ or utilizat$).tw.
258. (complie$ or comply or compliance$ or noncompliance$ or non compliance$).tw.
259. (encourag$ or discourage$ or reluctan$ or nonrespon$ or non respon$ or refuse$).tw.
260. (non-attend$ or non attend$ or dropout or drop out or apath$).tw.
261. Health Education/

For peer review only

262. exp Patient Education as Topic/

263. exp Health Promotion/

264. exp Counseling/

265. "Attitude of Health Personnel"/

266. (health adj2 (promotion$ or knowledge or belief$)).tw.

267. (educat$ adj2 (intervention$ or information or material or leaflet)).tw.

268. Socioeconomic Factors/

269. exp Poverty/

270. Social Class/

271. Educational Status/

272. ((school or education$) adj3 (status or level$ or attain$ or achieve$)).tw.

273. Employment/

274. Healthcare Disparities/

275. Health Status Disparities/

276. exp Medically Underserved Area/

277. Rural Population/

278. Urban Population/

279. exp Ethnic Groups/

280. Minority Groups/

281. Vulnerable Populations/

282. ((health$ or social$ or racial$ or ethnic$) adj5 (inequalit$ or inequit$ or disparit$ or equit$ or disadvantage$ or depriv$)).tw.

283. (disadvant$ or marginali$ or underserved or under served or impoverish$ or minorit$ or racial$ or ethnic$).tw.

284. (day adj3 (care or case$ or surger$)).tw.

285. (first eye adj1 cataract$).tw.

286. (second eye adj1 cataract$).tw.

287. (fellow eye adj1 cataract$).tw.

288. (simultaneous adj2 (phaco$ or phako$ or cataract$)).tw.

289. (bilateral adj2 (cataract$ surg$ or cataract$ extract$ or cataract$ remov$)).tw.

290. (sequential adj2 (cataract$ surg$ or cataract$ extract$ or cataract$ remov$)).tw.

291. Computer Simulation/

292. (virtual$ or simulat$).tw.

293. (residenc$ or resident$ or curriculum).tw.

For peer review only

294. or/129-293

295. 82 or 103 or 112 or 128 or 294

296. 4 and 295

297. epidemiologic studies/ or case-control studies/ or cohort studies/ or follow-up studies/ or longitudinal studies/ or prospective studies/ or controlled before-after studies/ or cross-sectional studies/ or historically controlled study/ or interrupted time series analysis/

298. epidemiologic methods/ or focus groups/ or interviews as topic/ or exp "surveys and questionnaires"/

299. epidemiologic research design/ or control groups/ or cross-over studies/ or double-blind method/ or meta-analysis as topic/ or network meta-analysis/ or random allocation/ or single-blind method/

300. epidemiologic methods/ or clinical trials as topic/ or feasibility studies/ or multicenter studies as topic/ or pilot projects/ or sampling studies/ or twin studies as topic/

301. randomized controlled trial/ or controlled clinical trials as topic/ or randomized controlled trials as topic/

302. comparative study/ or evaluation studies/ or meta-analysis/ or multicenter study/ or "systematic review"/ or validation studies/

303. Educational Measurement/

304. "Outcome and Process Assessment (Health Care)"/ or "Outcome Assessment (Health Care)"/

305. (cross adj1 section$).tw.

306. (cohort or intervention or prospective or comparative).tw.

307. (questionnaire$ or survey$).tw.

308. focus group$.tw.

309. (randomized or randomised or randomly).tw.

310. or/297-309

311. 296 and 310

312. (glaucoma$ or trabeculectom$ or angle closure or diabetic retinopath$ or keratoplast$ or keratopath$ or pseudoexfoliat$ or macula$ edema or macula$ oedema or retinal detachment$ or macula$ degeneration or scleral buckl$ or dry eye$ or uveitis or endothelial or endothelium or myopia or myopic or exotropia or amblyopia).ti.

313. (IOL$ or intraocular lens$ or trifocal or bifocal or multifocal or monofocal).ti.

314. (phacoemulsificat$ or capsulorhexis or wavefront or lensectomy or femtosecond or ECCE or SICS or MSICS or small incision or suture).ti.

For peer review only

315. (incidence or incident or prevalence).ti.

316. (dexamethasone or povidine or iodine or diclofenac or prednisolone or indomethacin or betaxolol or triamcinolone or nepafenac or corticosteroid$ or fluorouracil or bevacizumab or ranibizumab or radiation or ultrasound or intracameral or intravitreal or pseudophak$ or limbal or PMMA).ti.

317. optical coherence tomography.ti.

318. (genotyp$ or phenotyp$ or biomarker$ or genes or chromosome$ or mutation$).ti.

319. or/312-318

320. 311 not 319

321. exp case reports/

322. (case$ adj3 (report$ or stud$ or series)).tw.

323. 321 or 322

324. 320 not 323

325. limit 324 to (comment or editorial or letter or observational study)

326. 324 not 325

327. limit 326 to yr="1990 -Current"

For peer review only

BMJ Open

# Interventions to improve quality of cataract services: protocol for a scoping review

| | |
|---|---|
| Journal: | *BMJ Open* |
| Manuscript ID | bmjopen-2019-036413.R1 |
| Article Type: | Protocol |
| Date Submitted by the Author: | 17-Mar-2020 |
| Complete List of Authors: | Yoshizaki, Miho; London School of Hygiene and Tropical Medicine International Centre for Eye Health<br>Ramke, Jacqueline; London School of Hygiene and Tropical Medicine International Centre for Eye Health; The University of Auckland, School of Optometry and Vision Science<br>Furtado, João; Universidade de São Paulo Faculdade de Medicina de Ribeirão Preto, Division of Ophthalmology<br>Burn, Helen; Stoke Mandeville Hospital<br>Gichuhi, Stephen; University of Nairobi, Department of Ophthalmology<br>Gordon, Iris; London School of Hygiene and Tropical Medicine International Centre for Eye Health<br>Aghaji, Ada; University of Nigeria, Department of Ophthalmology<br>Marques, Ana Patricia; London School of Hygiene and Tropical Medicine International Centre for Eye Health<br>Dean, William; London School of Hygiene and Tropical Medicine International Centre for Eye Health; University of Cape Town, Department of Ophthalmology<br>Congdon, Nathan; Queen's University Belfast, Centre for Public Health; Sun Yat-Sen University, Zhongshan Ophthalmic Center<br>Buchan, John; London School of Hygiene and Tropical Medicine International Centre for Eye Health<br>Burton, Matthew J; London School of Hygiene and Tropical Medicine International Centre for Eye Health; Moorfields Eye Hospital |
| <b>Primary Subject Heading</b>: | Ophthalmology |
| Secondary Subject Heading: | Patient-centred medicine |
| Keywords: | Cataract and refractive surgery < OPHTHALMOLOGY, Quality in health care < HEALTH SERVICES ADMINISTRATION & MANAGEMENT, Health policy < HEALTH SERVICES ADMINISTRATION & MANAGEMENT |
| | |

SCHOLARONE™
Manuscripts

*I, the Submitting Author has the right to grant and does grant on behalf of all authors of the Work (as defined in the below author licence), an exclusive licence and/or a non-exclusive licence for contributions from authors who are: i) UK Crown employees; ii) where BMJ has agreed a CC-BY licence shall apply, and/or iii) in accordance with the terms applicable for US Federal Government officers or employees acting as part of their official duties; on a worldwide, perpetual, irrevocable, royalty-free basis to BMJ Publishing Group Ltd ("BMJ") its licensees and where the relevant Journal is co-owned by BMJ to the co-owners of the Journal, to publish the Work in this journal and any other BMJ products and to exploit all rights, as set out in our licence.*

*The Submitting Author accepts and understands that any supply made under these terms is made by BMJ to the Submitting Author unless you are acting as an employee on behalf of your employer or a postgraduate student of an affiliated institution which is paying any applicable article publishing charge ("APC") for Open Access articles. Where the Submitting Author wishes to make the Work available on an Open Access basis (and intends to pay the relevant APC), the terms of reuse of such Open Access shall be governed by a Creative Commons licence – details of these licences and which Creative Commons licence will apply to this Work are set out in our licence referred to above.*

*Other than as permitted in any relevant BMJ Author's Self Archiving Policies, I confirm this Work has not been accepted for publication elsewhere, is not being considered for publication elsewhere and does not duplicate material already published. I confirm all authors consent to publication of this Work and authorise the granting of this licence.*

For peer review only

# Interventions to improve quality of cataract services: protocol for a scoping review

Miho Yoshizaki[1], Jacqueline Ramke[1,2], João M. Furtado[3], Helen Burn[4], Stephen Gichuhi[5], Iris Gordon[1], Ada Aghaji[6], Ana Patricia Marques[1], William H Dean[1,7], Nathan Congdon[8,9], John Buchan[1], Matthew J Burton[1,10]

1. International Centre for Eye Health, London School of Hygiene & Tropical Medicine, London, United Kingdom
2. School of Optometry and Vision Science, University of Auckland, Auckland, New Zealand
3. Division of Ophthalmology, Ribeirão Preto Medical School, University of São Paulo, Ribeirão Preto, São Paulo, Brazil
4. Department of Ophthalmology, Stoke Mandeville Hospital, Aylesbury, United Kingdom
5. Department of Ophthalmology, University of Nairobi, Nairobi, Kenya
6. Department of Ophthalmology, College of Medicine, University of Nigeria, Nsukka, Nigeria
7. Department of Ophthalmology, University of Cape Town, Cape Town, South Africa
8. Centre for Public Health, Queens University Belfast, Belfast, UK
9. Zhongshan Ophthalmic Center, Sun Yat-sen University, Guangzhou, China
10. Moorfields Eye Hospital, London, United Kingdom

**Corresponding Author:** Miho Yoshizaki (miho.yoshizaki1@lshtm.ac.uk)

For peer review only



For peer review only - http://bmjopen.bmj.com/site/about/guidelines.xhtml

## ABSTRACT

### Introduction

Cataract is the leading cause of blindness globally, and a major cause of vision impairment. Cataract surgery is an efficacious intervention that usually restores vision. Although it is one of the most commonly conducted surgical interventions worldwide, good quality services (from being detected with operable cataract, to undergoing surgery and receiving post-operative care) are not universally available. Poor quality understandably reduces the willingness of people with operable cataract to undergo surgery. Therefore, it is critical to improve quality of care to subsequently reduce vision loss from cataract. This scoping review aims to summarise the nature and extent of the published literature on interventions to improve the quality of services for primary age-related cataract globally.

### Methods and analysis

We will search MEDLINE, Embase and Global Health for peer-reviewed manuscripts published since 1990, with no language, geographic or study design restrictions. To define quality, we have used the elements adopted by the World Health Organization—effectiveness, safety, people-centredness, timeliness, equity, integration and efficiency—to which we have added the element of planetary health. We will exclude studies focused on the technical aspects of the surgical procedure, and studies that only involve children (<18 years). Two reviewers will screen all titles/abstracts independently, followed by full-text review of potentially relevant articles. For included articles, data regarding publication characteristics, study details and quality related outcomes will be extracted by two reviewers independently. Results will be synthesised narratively and presented visually using a spider chart.

### Ethics and dissemination

Ethical approval is not required, as our review will only include published and publicly accessible information. We will publish our findings in an open-access peer-reviewed journal and develop an accessible summary of the results for website posting. A summary of the results will be included in the ongoing Lancet Global Health Commission on Global Eye Health.


**Registration details** https://osf.io/8gktz



For peer review only - http://bmjopen.bmj.com/site/about/guidelines.xhtml

**STRENGTHS AND LIMITATIONS OF THIS STUDY**

- In many settings, quality of cataract services is only measured by post-operative visual acuity. One of the strengths of this review is the use of a broader concept of quality which includes the seven elements outlined in WHO's framework for healthcare quality, as well as the element of planetary health.
- Another strength is that we have broadened the scope of cataract services beyond the surgical intervention itself, to identify elements of service delivery that can impact on the broad range of quality elements.
- We have excluded studies assessing specific surgical techniques and/or specific products and medications as we want to focus on interventions to improve detection, accessibility, uptake of surgery and overall outcome of the treatment across the service pathway. As such, we will exclude studies that report interventions involving intra-operative surgical and anaesthetic techniques, equipment and medication. This may be seen as a limitation by some, however this extensive literature is commonly synthesized in Cochrane and other reviews.
- A potential limitation is the paucity of published literature on interventions that address some elements of quality.



For peer review only - http://bmjopen.bmj.com/site/about/guidelines.xhtml

## INTRODUCTION

Cataract is the leading cause of blindness globally, and a major cause of moderate and severe vision impairment—an estimated 65 million people had vision loss from cataract in 2015.[1] Vision loss from cataract is unequally distributed throughout the world. For example, in 2015 among adults 50 years and above, the age standardized prevalence of cataract blindness ranged from 0.08% (80% uncertainty interval [UI] 0.03–0.19%) in high income countries of the Asia Pacific region to 2.35% (80% UI 0.72–5.04%) in West sub-Saharan Africa—almost a 30-fold difference.[1] Inequality (i.e. measurable differences between population subgroups) is also evident within countries, with a higher prevalence of cataract blindness among socially disadvantaged groups such as women, rural dwellers, and those who are not literate.[2]

Cataract surgery is an efficacious intervention that can restore vision[3-5] and alleviate poverty.[6] It is one of the most common surgical interventions in many high-income countries, and some middle-income countries.[7] However, good quality services are not universally available, particularly in low- and middle-income countries (LMICs).[8 9] Poor quality understandably reduces the willingness of people with operable cataract to undergo surgery.[10] Therefore it is critical to improve quality of care to subsequently reduce vision loss from cataract.

Quality of cataract services is most commonly measured using post-operative visual acuity. Measuring and monitoring outcomes is crucial in order to improve them[11] and tools are available to enable monitoring of post-operative visual acuity.[12]

Beyond using post-operative visual acuity to assess effectiveness, quality of cataract services includes many clinical and non-clinical dimensions.[13] For example:

- Timeliness: Cataract commonly occurs bilaterally. In many settings the current recommendation is to operate on one eye at a time and allow enough time for the operated eye to heal before operating on the second eye. However, delay in surgery for the second eye has been linked to increased risk of falls and road traffic accidents.[14]
- People-centredness: It may be common for patients to have to visit hospitals several times before the surgery for different pre-operative assessments, even though some of these could be done in one visit. Reducing the number of hospital visits to get surgery would improve quality from the patient perspective.
- Equity: There is no physiological reason why outcomes should be poorer in women compared to men, but women tend to have lower access and poorer post-operative vision outcomes



For peer review only - http://bmjopen.bmj.com/site/about/guidelines.xhtml

compared to men.[2 15] A further example of inequity is seen in the difference in effective cataract surgical coverage among Indigenous (51.6%, 95% confidence interval (CI) 42.4-60.7) and non-Indigenous Australians (88.5%, 95%CI 85.2-91.2).[16]

- Efficiency (Productivity): There is a link between the quantity of surgery a surgeon performs, and the quality of that surgery.[17] It has also been demonstrated that apparently cheaper service delivery options, such as outreach camps, can be less cost-effective compared to surgery delivered in static clinics due to worse outcomes.[18]

The aim of this review is to summarise the nature and extent of the published literature on interventions to improve the quality of cataract services globally. We chose to undertake a scoping review rather than an alternative evidence synthesis approach because we wished to identify and map the available evidence, which we anticipate will be heterogeneous.[19 20] We will take a broad perspective on quality outcomes and relevant interventions of interest, but will exclude studies focussed exclusively on the technical aspects of surgical techniques. For example, we will not include studies reporting effectiveness of phacoemulsification or manual small incision surgery, as these are summarised in other reviews.[3-5 21]

**Definitions and framework development**

Cataract services includes the range of activities on the pathway from detecting people with operable cataract, to these people undergoing surgery and receiving post-operative care. As such, cataract services are both community and facility-based,[22] and—regardless of the setting—should involve a broad range of health care providers from the community level (e.g. village health workers as case-finders) through primary (e.g. optometrist) and secondary services (i.e. surgical team). In addition, consideration of all of the health system building blocks is relevant to strengthen cataract services.

Quality-of-care is one of the objectives embodied by the concept of Universal Health Coverage (UHC), together with equity in access and financial protection.[23] Our review will be guided by the definition of quality of care recently outlined by World Health Organisation (WHO):

> *Quality of care* is *'the degree to which health services for individuals and populations increase the likelihood of desired health outcomes and are consistent with current professional knowledge'.*[24]



For peer review only - http://bmjopen.bmj.com/site/about/guidelines.xhtml

WHO has adopted the framework of quality outlined by the Institute of Medicine.[25] This framework measures quality of healthcare across seven elements, namely, effectiveness, safety, people-centredness, timeliness, equity, integration and efficiency.

We have made one addition to the quality elements in WHO's framework—we believe that *planetary health* is an essential element of quality cataract surgery, so will also scope the literature on this. Planetary health is focused on sustainability, including the ability of the society to make choices while balancing the needs of future generations.[26] This modified framework is shown in Figure 1.

To help guide the scope of our review, we mapped examples of outcome measures and interventions for cataract services against each of the eight elements of health care quality (Table 1). These outcomes and interventions were drawn from the literature,[27 28] as well as the knowledge and experience of the authorship group. For people-centredness, we drew on the outline of Integrated Person-Centred Health Services provided by WHO and adopted in the recent *World Report on Vision,* whereby services aim to provide coordinated care that addresses the full spectrum of eye conditions according to an individual's needs, and recognises people as participants and beneficiaries of this care.[29 30]

When mapping interventions, we categorised them using the WHO health systems "building blocks" i.e. we mapped them to the most relevant of Service delivery; Health workforce / Human Resources (HR), Health Information System (HIS); Access to essential consumables/non-consumables; Financing; and Leadership/governance. Recognising that this framework does not include community engagement and empowerment, we added *community* as an additional category against which interventions could be mapped.[31]



For peer review only - http://bmjopen.bmj.com/site/about/guidelines.xhtml

Table 1: Indicative outcomes and interventions to improve quality of cataract services*

| Quality elements | Description / notes | Example outcome measures | Example interventions |
|---|---|---|---|
| Effectiveness | WHO framework's defines this as adherence to evidence-based medicine.[24] | • Effective cataract surgical coverage[15]<br>• Pre and post-operative Visual Acuity (VA)<br>• Contrast, glare, colour vision<br>• Years of sight-loss avoided | • ***Service delivery:*** day case vs. in-patient surgery; risk stratification of patients and matching with surgeon skills<br>• ***Equipment/consumables:*** pre-operative biometry correctly undertaken and interpreted; access to good quality range of intraocular lens (IOL) powers<br>• ***HIS***: recording and monitoring of outcomes - national data reporting system i.e. cataract surgery minimum dataset in UK and annual audit based on this data[32]; PRECOG[33]; BOOST[12]; national benchmarks for quality outcomes; post-operative spectacle supply |
| Safety | Patient harm is the 14th leading cause of global disease burden.[24] | • Wrong lens insertion<br>• Post-operative issues e.g. endophthalmitis, cystoid macular oedema, retinal detachment, corneal oedema and decompensation incidents<br>• Refractive outcomes e.g. target spherical equivalent, prediction error, post-operative astigmatism | • ***Service delivery***: interventions to address surgical complications; protocols for emergency management of post-operative complications; post-operative care<br>• ***HR***: simulation training; continuing professional development for ophthalmologists<br>• ***HIS***: system to monitor individual surgeon performance;<br>• ***Governance:*** national benchmarks for quality outcomes in place (including refraction) quality assurance practice (i.e. WHO cataract check list, monitoring of outcomes)<br>• ***Equipment/consumables:*** IOL quality control, instrument sterilisation |
| People-centeredness | A good quality service should systematically incorporate needs and preferences of patients. | • Patient Reported Outcome Measures e.g. EQ-5D, HUI3, CatPROM5, Catquest-9SF, NEI VFQ-25<br>• Number of hospital attendances required | • ***Community:*** counselling about accessing surgery; informed consent process; social support (e.g. escort, family permission/support); dedicated eye health coordinators; pre-operative anxiety reduction strategies |
| Timeliness | Timely access to cataract surgery would improve patients experience and reduce the risk of complications. Early identification and appropriate referral is key to timely access. | • Severity of cataract at first presentation (including bilateral or unilateral)<br>• Time from diagnosis with operable cataract to completion of surgery<br>• Inter-operative time for patients with bilateral cataract | • ***Service delivery:*** re-design of pathways (diagnostics, referrals, treatment, follow-up) to be acceptable, affordable and sustainable; use of technology e.g. telemedicine; same-day bilateral surgery in low population density, low infection setting; strategies to reduce waiting list |
| Equity | Quality of care should not vary within a same setting according to patients' characteristics such | • Prevalence of cataract blindness and vision impairment (VI) in sub- | • ***Service delivery:*** outreach diagnostic protocols including consideration for false positives/negatives |



For peer review only - http://bmjopen.bmj.com/site/about/guidelines.xhtml

| | | | |
|---|---|---|---|
| | as age, gender, ethnicity, rural/urban and socio-economic status.<br><br>Equity can be considered in terms of equity of access to healthcare services or equity of health outcomes. | population (e.g. gender, ethnic minority, indigeneity)<br>• Volume, distribution and effective coverage of surgery in sub-populations | • ***Equipment/consumables:*** reduced tax on imported items<br>• ***Community:*** financial support for patients who need it (i.e. subsidy for surgery, transport); patient information and education to raise awareness/anxiety management<br>• ***Financing:*** health insurance for cataract surgery[34] |
| Integration | Continuity of care and care coordination, including coordinating care for effectively managing comorbidities<br>Improve the care experience for people | • Referral pathways<br>• Multidisciplinary team training, accreditation and governance structure | • ***Service delivery:*** pathways (diagnostics, treatment, follow-up); support service; outreach and primary care screening diagnostic protocols / algorithms including consideration for false positives/negatives |
| Efficiency | Efficient use of resources, including productivity of surgeons, would contribute to quality improvement at population level.<br><br>Health service efficiency can be considered as allocative efficiency (optimal mix of inputs is being used to produce chosen outputs i.e. multi-disciplinary team, financial allocation) and technical efficiency (i.e. productivity of surgeons etc.) | • Productivity of surgeons (i.e. annual cataract operations per surgeon)<br>• Availability of manager/administrator<br>• Multi-disciplinary fixed/permanent team<br>• Financial management<br>• Cost-effectiveness analysis | • ***HR***: multidisciplinary team to support the surgeon - e.g. nurses seeing post-operative patients; task-shifting to non-ophthalmologist cataract surgeons; eye department manager; removing the need for specialist anaesthetist<br>• ***Financing***: financial sustainability of the providers; eye department autonomy over funds (budget and/or bank account); payment options that incentivise productivity and quality improvement (i.e. fee per service, bundled payment); modelling of cost recovery options that balance productivity, affordability and profit<br>• ***Equipment/consumables***: dedicated operating theatre |
| Planetary Health | Healthcare is a major consumer of energy and resources and produces considerable amounts of emissions and waste. In order to protect and improve the health and wellbeing of future generations, it needs to shift towards environmentally sustainable system. | • Carbon footprint of cataract surgery<br>• Waste generated during cataract surgery | • ***Equipment/consumables***: reusable equipment, waste management<br>• ***HIS***: audit, lifecycle assessment<br>• ***Financing***: sustainable procurement |

*excluding surgical and aesthetical technical aspects, equipment and medication



For peer review only - http://bmjopen.bmj.com/site/about/guidelines.xhtml

# METHODS AND ANALYSIS

## Objectives / Scoping review questions

We aim to answer the following four questions:

1. What interventions to improve quality of cataract services have been described in the published literature?
2. Which element(s) of quality did the interventions address?
3. Where was the evidence generated (high- vs middle- vs low-income settings)?

## Protocol and registration

This protocol for this scoping review is reported according to the relevant sections of the PRISMA Extension for Scoping Reviews (PRISMA-ScR) guideline (Appendix 1).[35] The protocol is registered on the Open Science Framework (https://osf.io/8gktz).

## Eligibility criteria

This scoping review will include primary research studies of any design and systematic reviews from any country that report a quality-relevant outcome for primary age-related cataract following an intervention related to quality of cataract services. We will only include studies where an intervention is compared against any alternatives (e.g. intervention vs. no intervention / current practice vs. new intervention / before vs. after implementation). Examples of relevant interventions are provided in Table 1, mapped against the eight quality elements of interest. Systematic reviews will be included only if meta-analysis is conducted for a quality-relevant.

We will exclude studies assessing specific surgical techniques (e.g. phacoemulsification versus manual small incision surgery, site of anaesthesia, size of incision) and/or specific products and medications used during and around the time of surgery (e.g. monofocal versus multifocal intraocular lens, drug A versus drug B) as these are typically addressed in other systematic reviews.[3-5 21] Studies focussed exclusively on cataract services for children (aged under 18 years) will be excluded, as these services differ substantially from those for age-related cataract. We will also exclude studies reporting interventions to prevent cataract formation or progression. We will exclude studies published prior to 1990, as during the last 30 years there have been a large number of major developments in cataract services that would be expected to have changed the "landscape" substantially. Service delivery models prior to this time are quite different to those currently used. There will be no language limitations. Only studies where the full text is available will be included.



For peer review only - http://bmjopen.bmj.com/site/about/guidelines.xhtml

**Search**

We will search MEDLINE, Embase and Global Health databases using search strategies developed by a Cochrane Eyes and Vision Information Specialist (IG). The search strategy for MEDLINE is included in Appendix 2. We will examine reference lists of all included articles to identify further potentially relevant reports of studies. Field experts will be provided a list of the included studies and requested to identify further potentially relevant studies for consideration in the review.

**Selection of sources of evidence**

Covidence systematic review software will be used for screening (Veritas Health Innovation, Melbourne, Australia. Available at www.covidence.org). Each title and abstract will be screened independently by two reviewers (MY, JR, HB, AA, JB, JF, SG, WD) to exclude publications that clearly do not meet the inclusion criteria. Subsequently, the full text article will be retrieved for review if the citation seems potentially relevant and two reviewers will independently assess each article against the inclusion and exclusion criteria. Any discrepancies between the reviewers will be resolved by discussion, and a third reviewer will be consulted if necessary. A PRISMA flow diagram will be completed to summarise the study selection process.

**Data charting process**

A custom form will be developed in Excel for data charting. The form will be piloted on three studies and required amendments agreed by consensus. We anticipate a broad scope of included studies, so data charting will be an iterative process throughout the review and the data charting form will be amended as required. Each included study will be charted independently by two reviewers. Any discrepancies will be resolved by discussion, and a third reviewer will be consulted if necessary. We plan to contact study authors in the case of unclear information and will make up to three attempts by email.

**Data items**

The following data items will be collected during the data charting process:

1. Publication characteristics: title, year of publication, study design, country of origin, study setting;
2. Characteristics of intervention/study:



For peer review only - http://bmjopen.bmj.com/site/about/guidelines.xhtml

   a. Context (e.g. geographic area, target population and distribution, type of interventions (categorised by health system building block), target health practitioner, duration / frequency);
   b. Quality element(s) addressed by the intervention (as outlined in Table 1);
3. Outcome(s) of the intervention/study and whether it was reported to be effective (i.e. had an effect versus had no effect) (examples of outcomes outlined in Table 1).

**Synthesis of results**

We recognise that the indication for surgery can vary across different settings due to the prevalence of vision loss from cataract, the capacity of services and the quality and safety standards in each setting. Accordingly, we will synthesize results by World Bank country income-level (high / upper-middle / lower-middle / low)[36] and (if possible) by GBD Super-Region (High income / Latin America & Caribbean / Sub-Saharan Africa / North Africa & Middle East / Southeast Asia, East Asia & Oceania / South Asia / Central Europe, Eastern Europe & Central Asia).[37]

We will summarise findings narratively and using descriptive statistical methods as appropriate. We will map each intervention to the relevant quality element. We will visualise the findings using spider charts to show the extent of the evidence across each quality element and will plot evidence in high income countries separately to low- and middle-income countries. For each intervention, we will quantify the number of studies that were reported by the authors to be effective (versus having no effect).

**Patient and Public Involvement Statement**

This protocol was developed with input from the Commissioners of the *Lancet Global Health* Commission on Global Eye Health[38], which includes people with lived experience of vision impairment (and cataract surgery), policy makers, academics, clinicians, government eye health programme leaders and advocacy specialists.

**ETHICS AND DISSEMINATION**

Ethical approval is not required, as our review will only include published and publicly accessible information.

We will publish our findings in an open-access, peer-reviewed journal and develop an accessible summary of the results for website posting and stakeholder meetings. A summary of the results will also be included in the ongoing Lancet Commission on Global Eye Health.[38]



For peer review only - http://bmjopen.bmj.com/site/about/guidelines.xhtml

## References


1. Flaxman S, Bourne R, Resnikoff S, et al. Global Causes of Distance Vision Loss: 1990-2015 and projections to 2020. *Lancet Glob Health* 2017;5(12):e1221-e34.
2. Ramke J, Zwi AB, Lee AC, et al. Inequality in cataract blindness and services: moving beyond unidimensional analyses of social position *Br J Ophthalmol* 2017;101(4):395-400.
3. De Silva S, Riaz Y, Evans J. Phacoemulsification with posterior chamber intraocular lens versus extracapsular cataract extraction (ECCE) with posterior chamber intraocular lens for age-related cataract. *Cochrane Database Syst Rev* 2014(1)
4. De Silva S, Riaz Y, Evans J. Phacoemulsification with posterior chamber intraocular lens versus extracapsular cataract extraction (ECCE) with posterior chamber intraocular lens for age-related cataract. *Cochrane Database Syst Rev* 2014(1)
5. Riaz Y, De Silva S, Evans J. Manual small incision cataract surgery (MSICS) with posterior chamber intraocular lens versus phacoemulsification with posterior chamber intraocular lens for age-related cataract. 2013(10)
6. Kuper H, Polack S, Mathenge W, et al. Does cataract surgery alleviate poverty? Evidence from a multi-centre intervention study conducted in Kenya, the Philippines and Bangladesh. 2010;5(11):e15431.
7. Eurostat. Surgical operations and procedures statistics. Available from: https://ec.europa.eu/eurostat/statistics-explained/index.php?title=Surgical_operations_and_procedures_statistics (accessed 15 November 2019).
8. Zhao J, Xu X, Ellwein LB, et al. Cataract surgical coverage and visual acuity outcomes in rural China in 2014 and comparisons with the 2006 China Nine-Province Survey. *Am J Ophthalmol* 2018;193:62-70.
9. Silva JC, Mújica OJ, Vega E, et al. A comparative assessment of avoidable blindness and visual impairment in seven Latin American countries: prevalence, coverage, and inequality. *Rev Panam Salud Publica* 2015;37(10):13-20.
10. Yin Q, Hu A, Liang Y, et al. A two-site, population-based study of barriers to cataract surgery in rural China. *Invest Ophthalmol Vis Sci* 2009;50(3):1069-75.
11. Yorston D, Gichuhi S, Wood M, et al. Does prospective monitoring improve cataract surgery outcomes in Africa? *Br J Ophthalmol* 2002;86(5):543-47.
12. Congdon N, Suburaman G-B, Ravilla T, et al. Transforming research results into useful tools for global health: BOOST. *Lancet Glob Health* 2016;4(2):e96.
13. Donabedian A. The quality of care: how can it be assessed? *JAMA* 1988;260(12):1743-48.
14. Meuleners LB, Fraser ML, Ng J, et al. The impact of first-and second-eye cataract surgery on injurious falls that require hospitalisation: a whole-population study. *Age Ageing* 2013;43(3):341-46.
15. Ramke J, Gilbert C, Lee ACL, et al. Effective cataract surgical coverage: an indicator for measuring quality-of-care in the context of Universal Health Coverage *PLoS One* 2017;12(3):e0172342.
16. Keel S, Xie J, Foreman J, et al. Population-based assessment of visual acuity outcomes following cataract surgery in Australia: the National Eye Health Survey. *Br J Ophthalmol* 2018;102(10):1419-24.
17. Bell CM, Hatch WV, Cernat G, et al. Surgeon volumes and selected patient outcomes in cataract surgery: a population-based analysis. *Ophthalmology* 2007;114(3):405-10.
18. Singh A, Garner P, Floyd K. Cost-effectiveness of public-funded options for cataract surgery in Mysore, India. *Lancet* 2000;355(9199):180-84.
19. Joanna Briggs Institute. JBI Reviewer's manual. 2019. Available from: https://wiki.joannabriggs.org/display/MANUAL (accessed 9 March 2020).




For peer review only - http://bmjopen.bmj.com/site/about/guidelines.xhtml

20. Munn Z, Peters M, Stern C, et al. Systematic review or scoping review? Guidance for authors when choosing between a systematic or scoping review approach. *BMC Med Res Methodol* 2018;18(1):143.
21. Riaz Y, Mehta J, Wormald R, et al. Surgical interventions for age-related cataract. *Cochrane Database Syst Rev* 2006(4)
22. World Health Organization. Strategies for the prevention of blindness in national programmes: a primary health care approach (2nd ed). Geneva: World Health Organization 1997.
23. World Health Organization. What is universal coverage? 2016 Accessed 1 August 2017]. Available from: http://www.who.int/health_financing/universal_coverage_definition/en/).
24. World Health Organization. Delivering quality health services: a global imperative for universal health coverage. Geneva: WHO, 2018.
25. Institute of Medicine Committee on Health Care in America. Crossing the quality chasm: A new health system for the 21st century. Washington DC: National Academies Press 2001.
26. Health TLP. The bigger picture of planetary health. *Lancet Planet Health* 2019;3(1):e1.
27. Buchan J, Dean W, Foster A, et al. What are the priorities for improving cataract surgical outcomes in Africa? Results of a Delphi exercise. *Int Ophthalmol* 2018;38(4):1409-14.
28. Lindfield R, Vishwanath K, Ngounou F, et al. The challenges in improving outcome of cataract surgery in low and middle income countries. *Indian J Ophthalmol* 2012;60(5):464.
29. World Health Organization. Framework on integrated, people-centred health services. Geneva: WHO, 2016.
30. World Health Organization. World report on vision. Geneva: WHO, 2019.
31. Sacks E, Morrow M, Story WT, et al. Beyond the building blocks: integrating community roles into health systems frameworks to achieve health for all. *BMJ Glob Health* 2019;3(Suppl 3):e001384.
32. Day A, Donachie P, Sparrow J, et al. The Royal College of Ophthalmologists' National Ophthalmology Database study of cataract surgery: report 1, visual outcomes and complications. 2015;29(552-560)
33. Congdon N, Yan X, Lansingh V, et al. Assessment of cataract surgical outcomes in settings where follow-up is poor: PRECOG, a multicentre observational study. *Lancet Glob Health* 2013;1(1):e37-e45.
34. Ackuaku-Dogbe E, Yawson A, Biritwum R. Cataract surgical uptake among older adults in Ghana. *Ghana Med J* 2015;49(2):84-89.
35. Tricco AC, Lillie E, Zarin W, et al. PRISMA extension for scoping reviews (PRISMA-ScR): checklist and explanation. *Ann Intern Med* 2018;169(7):467-73.
36. World Bank. Country and lending groups. Available from: https://datahelpdesk.worldbank.org/knowledgebase/articles/906519-world-bank-country-and-lending-groups (accessed 2 November 2019).
37. Institute for Health Metrics and Evaluation. Frequently asked questions - what countries are in each regions? Available from: https://datahelpdesk.worldbank.org/knowledgebase/articles/906519-world-bank-country-and-lending-groups (accessed 15 November 2019).
38. Burton MJ, Faal HB, Ramke J, et al. Announcing The Lancet Global Health Commission on Global Eye Health. *Lancet Glob Health* 2019;7(12):e1612-e13.



For peer review only - http://bmjopen.bmj.com/site/about/guidelines.xhtml

**Authors' Contributions:** JR and MJB conceived the idea for the review. MY and JR drafted and revised the protocol with suggestions from MJB, NC, JMF, SG, HB, AA, APM, WD and JB. IG constructed the search.

**Corresponding Author:** Miho Yoshizaki (miho.yoshizaki1@lshtm.ac.uk)

**Funding Statement:** MJB is supported by the Wellcome Trust (207472/Z/17/Z). JR is a Commonwealth Rutherford Fellow, funded by the UK government through the Commonwealth Scholarship Commission in the UK. The Lancet Global Health Commission on Global Eye Health is supported by The Queen Elizabeth Diamond Jubilee Trust, Moorfields Eye Charity [grant number GR001061], NIHR Moorfields Biomedical Research Centre, Wellcome Trust, Sightsavers, The Fred Hollows Foundation, The SEVA Foundation, British Council for the Prevention of Blindness and Christian Blind Mission.

**Acknowledgements**:

**Competing Interests:** None declared

**Number of Tables**: 1

**Number of Figures:** 1

**Data Sharing Statement:** Data generated from this review will be available upon reasonable request from Jacqueline.Ramke@lshtm.ac.uk

**Keywords:** cataract, cataract services, quality, effectiveness, safety, person-centredness, timeliness, equity,

**Word Count: 2,018**

**Figure legend:**

Figure 1. Elements of health care quality considered in this review (modified Figure 3.2 from 'Delivering quality health services: a global imperative for universal health coverage'[24] by adding *Planetary Health*)

For peer review only

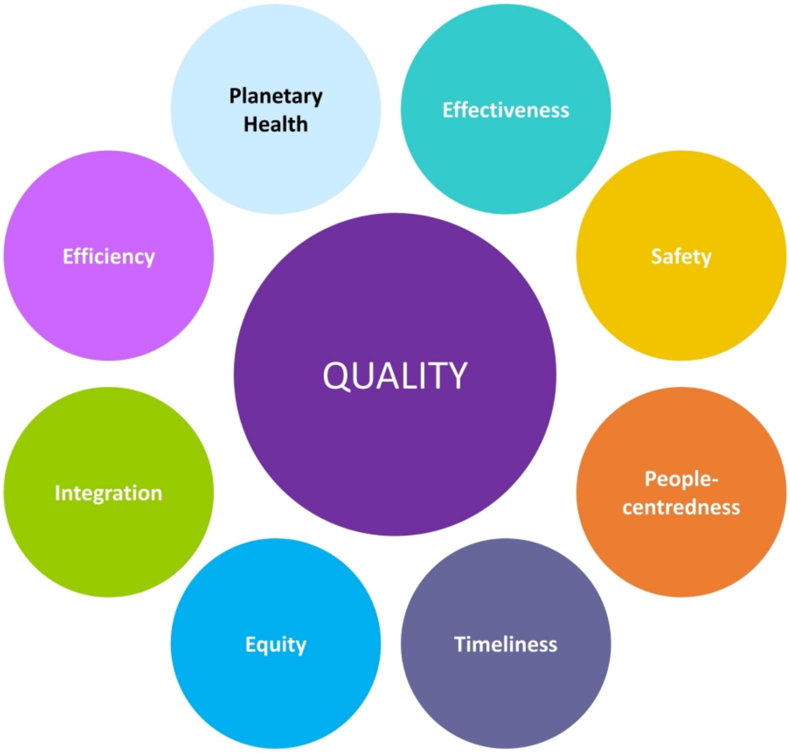


Figure 1. Elements of health care quality considered in this review (modified Figure 3.2 from 'Delivering quality health services: a global imperative for universal health coverage'24 by adding Planetary Health)

254x190mm (300 x 300 DPI)

## Annex 1: Preferred Reporting Items for Systematic reviews and Meta-Analyses extension for Scoping Reviews (PRISMA-ScR) Checklist

| SECTION | ITEM | PRISMA-ScR CHECKLIST ITEM | REPORTED ON PAGE # |
|---|---|---|---|
| **TITLE** | | | |
| Title | 1 | Identify the report as a scoping review. | 1 |
| **ABSTRACT** | | | |
| Structured summary | 2 | Provide a structured summary that includes (as applicable): background, objectives, eligibility criteria, sources of evidence, charting methods, results, and conclusions that relate to the review questions and objectives. | 2-3 |
| **INTRODUCTION** | | | |
| Rationale | 3 | Describe the rationale for the review in the context of what is already known. Explain why the review questions/objectives lend themselves to a scoping review approach. | 4-5 |
| Objectives | 4 | Provide an explicit statement of the questions and objectives being addressed with reference to their key elements (e.g., population or participants, concepts, and context) or other relevant key elements used to conceptualize the review questions and/or objectives. | 5-8 |
| **METHODS** | | | |
| Protocol and registration | 5 | Indicate whether a review protocol exists; state if and where it can be accessed (e.g., a Web address); and if available, provide registration information, including the registration number. | 9 |
| Eligibility criteria | 6 | Specify characteristics of the sources of evidence used as eligibility criteria (e.g., years considered, language, and publication status), and provide a rationale. | 9 |
| Information sources | 7 | Describe all information sources in the search (e.g., databases with dates of coverage and contact with authors to identify additional sources), as well as the date the most recent search was executed. | 9-10 |
| Search | 8 | Present the full electronic search strategy for at least 1 database, including any limits used, such that it could be repeated. | 9-10 |
| Selection of sources of evidence | 9 | State the process for selecting sources of evidence (i.e., screening and eligibility) included in the scoping review. | 10 |
| Data charting process | 10 | Describe the methods of charting data from the included sources of evidence (e.g., calibrated forms or forms that have been tested by the team before their use, and whether data charting was done independently or in duplicate) and any processes for obtaining and confirming data from investigators. | 10 |
| Data items | 11 | List and define all variables for which data were sought and any assumptions and simplifications made. | 10 |
| Critical appraisal of individual sources of evidence§ | 12 | If done, provide a rationale for conducting a critical appraisal of included sources of evidence; describe the methods used and how this information was used in any data synthesis (if appropriate). | N/A |
| Synthesis of results | 13 | Describe the methods of handling and summarizing the data that were charted. | 11 |

JBI = Joanna Briggs Institute; PRISMA-ScR = Preferred Reporting Items for Systematic reviews and Meta-Analyses extension for Scoping Reviews.

*From:* Tricco AC, Lillie E, Zarin W, O'Brien KK, Colquhoun H, Levac D, et al. PRISMA Extension for Scoping Reviews (PRISMA-ScR): Checklist and Explanation. Ann Intern Med. ;169:467–473. doi: 10.7326/M18-0850

For peer review only

**Annex 2: Example search - MEDLINE**

1. exp cataract/
2. Cataract Extraction/
3. cataract$.tw.
4. or/1-3
5. "Quality of Health Care"/
6. Quality Improvement/
7. Delivery of Health Care/
8. National Health Programs/
9. State Medicine/
10. Regional Health Planning/
11. Health Planning/
12. Health Plan Implementation/
13. Health Planning Guidelines/
14. Health Care Reform/
15. Health Resources/
16. Health Priorities/
17. Health Services Research/
18. "health services needs and demand"/
19. Needs Assessment/
20. State Health Plans/
21. Regional Health Planning/
22. Community Health Planning/
23. Hospital Planning/
24. Regional Medical Programs/
25. Health Maintenance Organizations/
26. Comprehensive Health Care/
27. Health Facility Planning/
28. Health Facility Administration/
29. Hospital Administration/
30. exp Hospitals, public/
31. exp Hospitals, private/
32. health system$.tw.

For peer review only

33. Models, Organizational/
34. Decision Making, Organizational/
35. Resource Allocation/
36. Efficiency, Organizational/
37. Organizational Innovation/
38. Delivery of Health Care, Integrated/
39. Interdisciplinary Communication/
40. Public Health/
41. Health Promotion/
42. Policy Making/
43. Program Development/
44. Program Evaluation/
45. Quality Control/
46. Quality Assurance, Health Care/
47. Benchmarking/
48. Capacity Building/
49. Health Services Accessibility/
50. Health Policy/
51. Surgical Procedures, Operative/
52. exp Surgical Equipment/
53. Health Care Rationing/
54. Medically Underserved Area/
55. Healthcare Disparities/
56. Health Status Disparities/
57. exp Attitude to Health/
58. "Patient Acceptance of Health Care"/
59. Health Education/
60. Public Opinion/
61. Health Behavior/
62. Social Behavior/
63. Superstitions/
64. exp Communication/
65. exp Culture/
66. Sex Factors/

For peer review only

67. Women's Rights/
68. Prejudice/
69. Vulnerable Populations/
70. Social Responsibility/
71. Social Welfare/
72. Urban Health Services/
73. Rural Health Services/
74. Rural Population/
75. (health adj20 (barrier$ or belie$ or inform$ or aware$ or knowledge or perceive$ or consequence$ or uptake or seek$ or underutili$ or fear$ or stigma$ or inequaliti$ or gender or logistic$ or distance$)).tw.
76. Patient Escort Service/
77. transport$.tw.
78. gender inequality.tw.
79. Mass Screening/
80. (referral adj3 (pathway$ or service$ or improve$)).tw.
81. (health worker$ or case finder$).tw.
82. or/5-81
83. Health Manpower/
84. Health Personnel/
85. Medical Staff, Hospital/
86. Nursing Staff, Hospital/
87. Personnel, Hospital/
88. Professional Competence/
89. Clinical Competence/
90. Medical Errors/
91. Professional Autonomy/
92. Leadership/
93. (leadership or motivat$).tw.
94. Motivation/
95. Organizational Innovation/
96. Personnel Selection/
97. Personnel Management/
98. Personnel Loyalty/

For peer review only

99. Job Satisfaction/
100. Staff Development/
101. "Attitude of Health Personnel"/
102. Personnel Turnover/
103. or/83-102
104. Clinical Governance/
105. Government Regulation/
106. Public Policy/
107. Public Health Practice/
108. Public Health Administration/
109. Health Plan Implementation/
110. Public-Private Sector Partnerships/
111. governance.tw.
112. or/104-111
113. (health management information system$ or HMIS).tw.
114. Management Information Systems/
115. Database Management Systems/
116. Computer Systems/
117. Point-of-Care Systems/
118. Hospital Information Systems/
119. Geographic Information Systems/
120. exp Medical Records Systems, Computerized/
121. Health Care Surveys/
122. Data Collection/
123. Data Interpretation, Statistical/
124. "Information Storage and Retrieval"/
125. Computer Literacy/
126. User-Computer Interface/
127. Attitude to Computers/
128. or/113-127
129. Delivery of Health Care, Integrated/
130. service delivery.tw.
131. decision making.tw.
132. (consensus adj3 (process$ or discuss)).tw.

For peer review only

133. stakeholder$.tw.
134. Quality Control/
135. Total Quality Management/
136. Quality Indicators, Health Care/
137. Quality Assurance, Health Care/
138. quality assurance.tw.
139. (quality adj2 improv$).tw.
140. total quality.tw.
141. continuous quality.tw.
142. quality management.tw.
143. (organisation$ adj3 cultur$).tw.
144. Disease Management/
145. Program Evaluation/
146. ((provider$ or program$) adj3 (monitor$ or evaluate$ or modif$ or practice)).tw.
147. (implement$ adj3 (improve$ or change$ or effort$ or issue$ or impede$ or glossary or tool$ or innovation$ or outcome$ or driv$ or examin$ or reexamin$ or scale$ or strateg$ or advis$ or expert$)).tw.
148. (needs adj3 assess$).tw.
149. ((education$ or learn$) adj5 (continu$ or material$ or meeting or collaborat$)).tw.
150. exp Medical audit/
151. (audit or feedback or compliance or adherence or training or innovation).ti.
152. (guideline$ adj3 (clinical or practice or implement$ or promot$)).tw.
153. exp Health Services Accessibility/
154. (outreach adj2 (service$ or visit$)).tw.
155. (intervention$ adj3 (no or usual or routine or target$ or tailor$ or mediat$)).tw.
156. usual care.tw.
157. exp Reminder Systems/
158. remind$.tw.
159. (improve$ adj3 (attend$ or visit$ or intervention$ or adhere$)).tw.
160. (increas$ adj3 (attend$ or visit$ or intervention$ or adhere$)).tw.
161. (appointment$ adj3 (miss$ or fail$ or remind$ or follow up)).tw.
162. Telephone/
163. telephone.tw.
164. Cell Phones/

For peer review only

165. Mobile Applications/
166. Remote Consultation/
167. (m-health or e-health or g-health or u-health).tw.
168. (phone$ adj1 (smart or cell)).tw.
169. (smartphone$ or cellphone$).tw.
170. (hand adj1 held device$).tw.
171. (mobile adj2 (health or healthcare or phone$ or device$ or monitor$ or comput$ or app or apps or application)).tw.
172. Internet/
173. Social Networking/
174. (email$ or text$ or message$).tw.
175. (letter or mail or mailed or print$ or brochure$ or newsletter$).tw.
176. Primary Health Care/
177. General Practitioners/ or Physicians, Family/ or Physicians, Primary Care/
178. Primary Prevention/
179. Preventive Health Services/
180. Community Health Services/
181. Community Health Nursing/
182. Health Services, Indigenous/
183. Rural Health Services/
184. Mobile Health Units/
185. (Ophthalmologist$ or Optometrist$ or Optician$ or Orthopist$ or Refractionists).tw.
186. ((Ophthalmic or eye) adj3 (surgeon$ or nurse$ or technician$ or officer$ or assistant$ or staff$)).tw.
187. Physician's Practice Patterns/
188. Professional Practice/
189. (professional adj3 (practice or develop$ or educat)).tw.
190. Education, Medical, Continuing/
191. exp nurses/
192. Specialties, Nursing/
193. Nurse's Role/
194. Education, Nursing, Continuing/
195. (nurse or nurses).tw.
196. Pharmacists/

For peer review only

197. pharmacist$.tw.
198. ((role or roles) adj3 expan$).tw.
199. (task$ adj3 shift$).tw.
200. exp Medical Records Systems, Computerized/
201. Management Information Systems/
202. Database Management Systems/
203. Computer Systems/
204. Point-of-Care Systems/
205. Hospital Information Systems/
206. ((health or healthcare) adj4 (record or management system$)).tw.
207. (decision adj5 support).ti.
208. Economics/
209. "costs and cost analysis"/
210. Cost allocation/
211. Cost-benefit analysis/
212. Cost control/
213. Cost savings/
214. Cost of illness/
215. Cost sharing/
216. "deductibles and coinsurance"/
217. Medical savings accounts/
218. Health care costs/
219. Direct service costs/
220. Drug costs/
221. Employer health costs/
222. Hospital costs/
223. Health expenditures/
224. Capital expenditures/
225. Value of life/
226. exp economics, hospital/
227. exp economics, medical/
228. Economics, nursing/
229. Economics, pharmaceutical/
230. exp "fees and charges"/

For peer review only

231. exp budgets/

232. (low adj cost).mp.

233. (high adj cost).mp.

234. (health?care adj cost$).mp.

235. (fiscal or funding or financial or finance).tw.

236. (cost adj estimate$).mp.

237. (cost adj variable).mp.

238. (unit adj cost$).mp.

239. (economic$ or pharmacoeconomic$ or price$ or pricing).tw.

240. Uncompensated Care/

241. Reimbursement Mechanisms/

242. Reimbursement, Incentive/

243. (insurance adj3 (health$ or scheme$)).tw.

244. (financial or economic or pay or payment or copayment or paid or fee or fees or monetary or money or cash or incentiv$ or disincentiv$).tw.

245. ((pay or paying or paid or cost$ or free or wait$ or qualit$) adj3 surg$).tw.

246. (will$ adj3 pay$).tw.

247. (waiting adj2 time).tw.

248. ((surgery or surgical) adj2 (experience or supervis$ or rate or rates or output or volume or uptake)).tw.

249. productivity.tw.

250. (patient adj3 (knowledge or satisfi$ or attitude$)).tw.

251. (percept$ adj3 quality).tw.

252. (follow up adj3 (appointment$ or poor or compliant or compliance)).tw.

253. exp Patient Acceptance of health Care/

254. exp Attitude to Health/

255. exp Health Behavior/

256. (barrier$ or obstacle$ or facilitat$ or enable$).tw.

257. (uptake or takeup or attend$ or accept$ or adhere$ or attitude$ or participat$ or facilitat$ or utilisat$ or utilizat$).tw.

258. (complie$ or comply or compliance$ or noncompliance$ or non compliance$).tw.

259. (encourag$ or discourage$ or reluctan$ or nonrespon$ or non respon$ or refuse$).tw.

260. (non-attend$ or non attend$ or dropout or drop out or apath$).tw.

261. Health Education/

For peer review only

262. exp Patient Education as Topic/
263. exp Health Promotion/
264. exp Counseling/
265. "Attitude of Health Personnel"/
266. (health adj2 (promotion$ or knowledge or belief$)).tw.
267. (educat$ adj2 (intervention$ or information or material or leaflet)).tw.
268. Socioeconomic Factors/
269. exp Poverty/
270. Social Class/
271. Educational Status/
272. ((school or education$) adj3 (status or level$ or attain$ or achieve$)).tw.
273. Employment/
274. Healthcare Disparities/
275. Health Status Disparities/
276. exp Medically Underserved Area/
277. Rural Population/
278. Urban Population/
279. exp Ethnic Groups/
280. Minority Groups/
281. Vulnerable Populations/
282. ((health$ or social$ or racial$ or ethnic$) adj5 (inequalit$ or inequit$ or disparit$ or equit$ or disadvantage$ or depriv$)).tw.
283. (disadvant$ or marginali$ or underserved or under served or impoverish$ or minorit$ or racial$ or ethnic$).tw.
284. (day adj3 (care or case$ or surger$)).tw.
285. (first eye adj1 cataract$).tw.
286. (second eye adj1 cataract$).tw.
287. (fellow eye adj1 cataract$).tw.
288. (simultaneous adj2 (phaco$ or phako$ or cataract$)).tw.
289. (bilateral adj2 (cataract$ surg$ or cataract$ extract$ or cataract$ remov$)).tw.
290. (sequential adj2 (cataract$ surg$ or cataract$ extract$ or cataract$ remov$)).tw.
291. Computer Simulation/
292. (virtual$ or simulat$).tw.
293. (residenc$ or resident$ or curriculum).tw.

For peer review only

294. or/129-293

295. 82 or 103 or 112 or 128 or 294

296. 4 and 295

297. epidemiologic studies/ or case-control studies/ or cohort studies/ or follow-up studies/ or longitudinal studies/ or prospective studies/ or controlled before-after studies/ or cross-sectional studies/ or historically controlled study/ or interrupted time series analysis/

298. epidemiologic methods/ or focus groups/ or interviews as topic/ or exp "surveys and questionnaires"/

299. epidemiologic research design/ or control groups/ or cross-over studies/ or double-blind method/ or meta-analysis as topic/ or network meta-analysis/ or random allocation/ or single-blind method/

300. epidemiologic methods/ or clinical trials as topic/ or feasibility studies/ or multicenter studies as topic/ or pilot projects/ or sampling studies/ or twin studies as topic/

301. randomized controlled trial/ or controlled clinical trials as topic/ or randomized controlled trials as topic/

302. comparative study/ or evaluation studies/ or meta-analysis/ or multicenter study/ or "systematic review"/ or validation studies/

303. Educational Measurement/

304. "Outcome and Process Assessment (Health Care)"/ or "Outcome Assessment (Health Care)"/

305. (cross adj1 section$).tw.

306. (cohort or intervention or prospective or comparative).tw.

307. (questionnaire$ or survey$).tw.

308. focus group$.tw.

309. (randomized or randomised or randomly).tw.

310. or/297-309

311. 296 and 310

312. (glaucoma$ or trabeculectom$ or angle closure or diabetic retinopath$ or keratoplast$ or keratopath$ or pseudoexfoliat$ or macula$ edema or macula$ oedema or retinal detachment$ or macula$ degeneration or scleral buckl$ or dry eye$ or uveitis or endothelial or endothelium or myopia or myopic or exotropia or amblyopia).ti.

313. (IOL$ or intraocular lens$ or trifocal or bifocal or multifocal or monofocal).ti.

314. (phacoemulsificat$ or capsulorhexis or wavefront or lensectomy or femtosecond or ECCE or SICS or MSICS or small incision or suture).ti.

For peer review only

315. (incidence or incident or prevalence).ti.

316. (dexamethasone or povidine or iodine or diclofenac or prednisolone or indomethacin or betaxolol or triamcinolone or nepafenac or corticosteroid$ or fluorouracil or bevacizumab or ranibizumab or radiation or ultrasound or intracameral or intravitreal or pseudophak$ or limbal or PMMA).ti.

317. optical coherence tomography.ti.

318. (genotyp$ or phenotyp$ or biomarker$ or genes or chromosome$ or mutation$).ti.

319. or/312-318

320. 311 not 319

321. exp case reports/

322. (case$ adj3 (report$ or stud$ or series)).tw.

323. 321 or 322

324. 320 not 323

325. limit 324 to (comment or editorial or letter or observational study)

326. 324 not 325

327. limit 326 to yr="1990 -Current"

For peer review only

BMJ Open

# Interventions to improve quality of cataract services: protocol for a global scoping review



| | |
|---|---|
| Journal: | *BMJ Open* |
| Manuscript ID | bmjopen-2019-036413.R2 |
| Article Type: | Protocol |
| Date Submitted by the Author: | 15-Apr-2020 |



| | |
|---|---|
| Complete List of Authors: | Yoshizaki, Miho; London School of Hygiene and Tropical Medicine International Centre for Eye Health<br>Ramke, Jacqueline; London School of Hygiene and Tropical Medicine International Centre for Eye Health; The University of Auckland, School of Optometry and Vision Science<br>Furtado, João; Universidade de São Paulo Faculdade de Medicina de Ribeirão Preto, Division of Ophthalmology<br>Burn, Helen; Stoke Mandeville Hospital<br>Gichuhi, Stephen; University of Nairobi, Department of Ophthalmology<br>Gordon, Iris; London School of Hygiene and Tropical Medicine International Centre for Eye Health<br>Aghaji, Ada; University of Nigeria, Department of Ophthalmology<br>Marques, Ana Patricia; London School of Hygiene and Tropical Medicine International Centre for Eye Health<br>Dean, William; London School of Hygiene and Tropical Medicine International Centre for Eye Health; University of Cape Town, Department of Ophthalmology<br>Congdon, Nathan; Queen's University Belfast, Centre for Public Health; Sun Yat-Sen University, Zhongshan Ophthalmic Center<br>Buchan, John; London School of Hygiene and Tropical Medicine International Centre for Eye Health<br>Burton, Matthew J; London School of Hygiene and Tropical Medicine International Centre for Eye Health; Moorfields Eye Hospital |



| | |
|---|---|
| <b>Primary Subject Heading</b>: | Ophthalmology |
| Secondary Subject Heading: | Patient-centred medicine |
| Keywords: | Cataract and refractive surgery < OPHTHALMOLOGY, Quality in health care < HEALTH SERVICES ADMINISTRATION & MANAGEMENT, Health policy < HEALTH SERVICES ADMINISTRATION & MANAGEMENT |
| | |


SCHOLARONE™
Manuscripts

*I, the Submitting Author has the right to grant and does grant on behalf of all authors of the Work (as defined in the below author licence), an exclusive licence and/or a non-exclusive licence for contributions from authors who are: i) UK Crown employees; ii) where BMJ has agreed a CC-BY licence shall apply, and/or iii) in accordance with the terms applicable for US Federal Government officers or employees acting as part of their official duties; on a worldwide, perpetual, irrevocable, royalty-free basis to BMJ Publishing Group Ltd ("BMJ") its licensees and where the relevant Journal is co-owned by BMJ to the co-owners of the Journal, to publish the Work in this journal and any other BMJ products and to exploit all rights, as set out in our licence.*

*The Submitting Author accepts and understands that any supply made under these terms is made by BMJ to the Submitting Author unless you are acting as an employee on behalf of your employer or a postgraduate student of an affiliated institution which is paying any applicable article publishing charge ("APC") for Open Access articles. Where the Submitting Author wishes to make the Work available on an Open Access basis (and intends to pay the relevant APC), the terms of reuse of such Open Access shall be governed by a Creative Commons licence – details of these licences and which Creative Commons licence will apply to this Work are set out in our licence referred to above.*

*Other than as permitted in any relevant BMJ Author's Self Archiving Policies, I confirm this Work has not been accepted for publication elsewhere, is not being considered for publication elsewhere and does not duplicate material already published. I confirm all authors consent to publication of this Work and authorise the granting of this licence.*

For peer review only

# Interventions to improve quality of cataract services: protocol for a global scoping review

Miho Yoshizaki[1], Jacqueline Ramke[1,2], João M. Furtado[3], Helen Burn[4], Stephen Gichuhi[5], Iris Gordon[1], Ada Aghaji[6], Ana Patricia Marques[1], William H Dean[1,7], Nathan Congdon[8,9], John Buchan[1], Matthew J Burton[1,10]

1. International Centre for Eye Health, London School of Hygiene & Tropical Medicine, London, United Kingdom
2. School of Optometry and Vision Science, University of Auckland, Auckland, New Zealand
3. Division of Ophthalmology, Ribeirão Preto Medical School, University of São Paulo, Ribeirão Preto, São Paulo, Brazil
4. Department of Ophthalmology, Stoke Mandeville Hospital, Aylesbury, United Kingdom
5. Department of Ophthalmology, University of Nairobi, Nairobi, Kenya
6. Department of Ophthalmology, College of Medicine, University of Nigeria, Nsukka, Nigeria
7. Department of Ophthalmology, University of Cape Town, Cape Town, South Africa
8. Centre for Public Health, Queens University Belfast, Belfast, UK
9. Zhongshan Ophthalmic Center, Sun Yat-sen University, Guangzhou, China
10. Moorfields Eye Hospital, London, United Kingdom

**Corresponding Author:** Miho Yoshizaki (miho.yoshizaki1@lshtm.ac.uk)

For peer review only



For peer review only - http://bmjopen.bmj.com/site/about/guidelines.xhtml

## ABSTRACT

### Introduction

Cataract is the leading cause of blindness globally, and a major cause of vision impairment. Cataract surgery is an efficacious intervention that usually restores vision. Although it is one of the most commonly conducted surgical interventions worldwide, good quality services (from being detected with operable cataract, to undergoing surgery and receiving post-operative care) are not universally available. Poor quality understandably reduces the willingness of people with operable cataract to undergo surgery. Therefore, it is critical to improve quality of care to subsequently reduce vision loss from cataract. This scoping review aims to summarise the nature and extent of the published literature on interventions to improve the quality of services for primary age-related cataract globally.

### Methods and analysis

We will search MEDLINE, Embase and Global Health for peer-reviewed manuscripts published since 1990, with no language, geographic or study design restrictions. To define quality, we have used the elements adopted by the World Health Organization—effectiveness, safety, people-centredness, timeliness, equity, integration and efficiency—to which we have added the element of planetary health. We will exclude studies focused on the technical aspects of the surgical procedure, and studies that only involve children (<18 years). Two reviewers will screen all titles/abstracts independently, followed by full-text review of potentially relevant articles. For included articles, data regarding publication characteristics, study details and quality related outcomes will be extracted by two reviewers independently. Results will be synthesised narratively and presented visually using a spider chart.

### Ethics and dissemination

Ethical approval is not required, as our review will only include published and publicly accessible information. We will publish our findings in an open-access peer-reviewed journal and develop an accessible summary of the results for website posting. A summary of the results will be included in the ongoing Lancet Global Health Commission on Global Eye Health.


**Registration details** https://osf.io/8gktz



For peer review only - http://bmjopen.bmj.com/site/about/guidelines.xhtml

## STRENGTHS AND LIMITATIONS OF THIS STUDY

- A strength of this review is the use of a broader concept of quality beyond the common measure of post-operative visual acuity – we included the seven elements of quality outlined in WHO's framework for healthcare quality, as well as the element of planetary health.
- Another strength is that we have broadened the scope of cataract services beyond the surgical intervention itself, to identify interventions to improve quality along the care pathways, from detection and referral to uptake of services through to post-operative care.
- This study will not include studies that assess specific surgical techniques and/or specific products and medications as this extensive literature is commonly synthesized in Cochrane and other reviews.
- This review will summarise the nature and extent of the literature on interventions to improve quality of cataract services but will not assess the quality or risk of bias of the studies themselves.

For peer review only

## INTRODUCTION

Cataract is the leading cause of blindness globally, and a major cause of moderate and severe vision impairment—an estimated 65 million people had vision loss from cataract in 2015.[1] Vision loss from cataract is unequally distributed throughout the world. For example, in 2015 among adults 50 years and above, the age standardized prevalence of cataract blindness ranged from 0.08% (80% uncertainty interval [UI] 0.03–0.19%) in high income countries of the Asia Pacific region to 2.35% (80% UI 0.72–5.04%) in West sub-Saharan Africa—almost a 30-fold difference.[1] Inequality (i.e. measurable differences between population subgroups) is also evident within countries, with a higher prevalence of cataract blindness among socially disadvantaged groups such as women, rural dwellers, and those who are not literate.[2]

Cataract surgery is an efficacious intervention that can restore vision[3-5] and alleviate poverty.[6] It is one of the most common surgical interventions in many high-income countries, and some middle-income countries.[7] However, good quality services are not universally available, particularly in low- and middle-income countries (LMICs).[8 9] Poor quality understandably reduces the willingness of people with operable cataract to undergo surgery.[10] Therefore it is critical to improve quality of care to subsequently reduce vision loss from cataract.

Quality of cataract services is most commonly measured using post-operative visual acuity. Measuring and monitoring outcomes is crucial in order to improve them[11] and tools are available to enable monitoring of post-operative visual acuity.[12]

Beyond using post-operative visual acuity to assess effectiveness, quality of cataract services includes many clinical and non-clinical dimensions.[13] For example:

- Timeliness: Cataract commonly occurs bilaterally. In many settings the current recommendation is to operate on one eye at a time and allow enough time for the operated eye to heal before operating on the second eye. However, delay in surgery for the second eye has been linked to increased risk of falls and road traffic accidents.[14]
- People-centredness: It may be common for patients to have to visit hospitals several times before the surgery for different pre-operative assessments, even though some of these could be done in one visit. Reducing the number of hospital visits to get surgery would improve quality from the patient perspective.
- Equity: There is no physiological reason why outcomes should be poorer in women compared to men, but women tend to have lower access and poorer post-operative vision outcomes



For peer review only - http://bmjopen.bmj.com/site/about/guidelines.xhtml

compared to men.[2 15] A further example of inequity is seen in the difference in effective cataract surgical coverage among Indigenous (51.6%, 95% confidence interval (CI) 42.4-60.7) and non-Indigenous Australians (88.5%, 95%CI 85.2-91.2).[16]

- Efficiency (Productivity): There is a link between the quantity of surgery a surgeon performs, and the quality of that surgery.[17] It has also been demonstrated that apparently cheaper service delivery options, such as outreach camps, can be less cost-effective compared to surgery delivered in static clinics due to worse outcomes.[18]

The aim of this review is to summarise the nature and extent of the published literature on interventions to improve the quality of cataract services globally. We chose to undertake a scoping review rather than an alternative evidence synthesis approach because we wished to identify and map the available evidence, which we anticipate will be heterogeneous.[19 20] We will take a broad perspective on quality outcomes and relevant interventions of interest, but will exclude studies focussed exclusively on the technical aspects of surgical techniques. For example, we will not include studies reporting effectiveness of phacoemulsification or manual small incision surgery, as these are summarised in other reviews.[3-5 21]

**Definitions and framework development**

Cataract services includes the range of activities on the pathway from detecting people with operable cataract, to these people undergoing surgery and receiving post-operative care. As such, cataract services are both community and facility-based,[22] and—regardless of the setting—should involve a broad range of health care providers from the community level (e.g. village health workers as case-finders) through primary (e.g. optometrist) and secondary services (i.e. surgical team). In addition, consideration of all of the health system building blocks is relevant to strengthen cataract services.

Quality-of-care is one of the objectives embodied by the concept of Universal Health Coverage (UHC), together with equity in access and financial protection.[23] Our review will be guided by the definition of quality of care recently outlined by World Health Organisation (WHO):

> *Quality of care* is *'the degree to which health services for individuals and populations increase the likelihood of desired health outcomes and are consistent with current professional knowledge'.*[24]



For peer review only - http://bmjopen.bmj.com/site/about/guidelines.xhtml

WHO has adopted the framework of quality outlined by the Institute of Medicine.[25] This framework measures quality of healthcare across seven elements, namely, effectiveness, safety, people-centredness, timeliness, equity, integration and efficiency.

We have made one addition to the quality elements in WHO's framework—we believe that *planetary health* is an essential element of quality cataract surgery, so will also scope the literature on this. Planetary health is focused on sustainability, including the ability of the society to make choices while balancing the needs of future generations.[26] This modified framework is shown in Figure 1.

To help guide the scope of our review, we mapped examples of outcome measures and interventions for cataract services against each of the eight elements of health care quality (Table 1). These outcomes and interventions were drawn from the literature,[27 28] as well as the knowledge and experience of the authorship group. For people-centredness, we drew on the outline of Integrated Person-Centred Health Services provided by WHO and adopted in the recent *World Report on Vision,* whereby services aim to provide coordinated care that addresses the full spectrum of eye conditions according to an individual's needs, and recognises people as participants and beneficiaries of this care.[29 30]

When mapping interventions, we categorised them using the WHO health systems "building blocks" i.e. we mapped them to the most relevant of Service delivery; Health workforce / Human Resources (HR), Health Information System (HIS); Access to essential consumables/non-consumables; Financing; and Leadership/governance. Recognising that this framework does not include community engagement and empowerment, we added *community* as an additional category against which interventions could be mapped.[31]

For peer review only

Table 1: Indicative outcomes and interventions to improve quality of cataract services*

| Quality elements | Description / notes | Example outcome measures | Example interventions |
|---|---|---|---|
| Effectiveness | WHO framework's defines this as adherence to evidence-based medicine.[24] | • Effective cataract surgical coverage[15]<br>• Pre and post-operative Visual Acuity (VA)<br>• Contrast, glare, colour vision<br>• Years of sight-loss avoided | • ***Service delivery:*** day case vs. in-patient surgery; risk stratification of patients and matching with surgeon skills<br>• ***Equipment/consumables:*** pre-operative biometry correctly undertaken and interpreted; access to good quality range of intraocular lens (IOL) powers<br>• ***HIS***: recording and monitoring of outcomes - national data reporting system i.e. cataract surgery minimum dataset in UK and annual audit based on this data[32]; PRECOG[33]; BOOST[12]; national benchmarks for quality outcomes; post-operative spectacle supply |
| Safety | Patient harm is the 14th leading cause of global disease burden.[24] | • Wrong lens insertion<br>• Post-operative issues e.g. endophthalmitis, cystoid macular oedema, retinal detachment, corneal oedema and decompensation incidents<br>• Refractive outcomes e.g. target spherical equivalent, prediction error, post-operative astigmatism | • ***Service delivery***: interventions to address surgical complications; protocols for emergency management of post-operative complications; post-operative care<br>• ***HR***: simulation training; continuing professional development for ophthalmologists<br>• ***HIS***: system to monitor individual surgeon performance;<br>• ***Governance:*** national benchmarks for quality outcomes in place (including refraction) quality assurance practice (i.e. WHO cataract check list, monitoring of outcomes)<br>• ***Equipment/consumables:*** IOL quality control, instrument sterilisation |
| People-centeredness | A good quality service should systematically incorporate needs and preferences of patients. | • Patient Reported Outcome Measures e.g. EQ-5D, HUI3, CatPROM5, Catquest-9SF, NEI VFQ-25<br>• Number of hospital attendances required | • ***Community:*** counselling about accessing surgery; informed consent process; social support (e.g. escort, family permission/support); dedicated eye health coordinators; pre-operative anxiety reduction strategies |
| Timeliness | Timely access to cataract surgery would improve patients experience and reduce the risk of complications. Early identification and appropriate referral is key to timely access. | • Severity of cataract at first presentation (including bilateral or unilateral)<br>• Time from diagnosis with operable cataract to completion of surgery<br>• Inter-operative time for patients with bilateral cataract | • ***Service delivery:*** re-design of pathways (diagnostics, referrals, treatment, follow-up) to be acceptable, affordable and sustainable; use of technology e.g. telemedicine; same-day bilateral surgery in low population density, low infection setting; strategies to reduce waiting list |
| Equity | Quality of care should not vary within a same setting according to patients' characteristics such | • Prevalence of cataract blindness and vision impairment (VI) in sub- | • ***Service delivery:*** outreach diagnostic protocols including consideration for false positives/negatives |



For peer review only - http://bmjopen.bmj.com/site/about/guidelines.xhtml

| | | | |
|---|---|---|---|
| | as age, gender, ethnicity, rural/urban and socio-economic status.<br><br>Equity can be considered in terms of equity of access to healthcare services or equity of health outcomes. | population (e.g. gender, ethnic minority, indigeneity)<br>• Volume, distribution and effective coverage of surgery in sub-populations | • ***Equipment/consumables:*** reduced tax on imported items<br>• ***Community:*** financial support for patients who need it (i.e. subsidy for surgery, transport); patient information and education to raise awareness/anxiety management<br>• ***Financing:*** health insurance for cataract surgery[34] |
| Integration | Continuity of care and care coordination, including coordinating care for effectively managing comorbidities<br>Improve the care experience for people | • Referral pathways<br>• Multidisciplinary team training, accreditation and governance structure | • ***Service delivery:*** pathways (diagnostics, treatment, follow-up); support service; outreach and primary care screening diagnostic protocols / algorithms including consideration for false positives/negatives |
| Efficiency | Efficient use of resources, including productivity of surgeons, would contribute to quality improvement at population level.<br><br>Health service efficiency can be considered as allocative efficiency (optimal mix of inputs is being used to produce chosen outputs i.e. multi-disciplinary team, financial allocation) and technical efficiency (i.e. productivity of surgeons etc.) | • Productivity of surgeons (i.e. annual cataract operations per surgeon)<br>• Availability of manager/administrator<br>• Multi-disciplinary fixed/permanent team<br>• Financial management<br>• Cost-effectiveness analysis | • ***HR***: multidisciplinary team to support the surgeon - e.g. nurses seeing post-operative patients; task-shifting to non-ophthalmologist cataract surgeons; eye department manager; removing the need for specialist anaesthetist<br>• ***Financing***: financial sustainability of the providers; eye department autonomy over funds (budget and/or bank account); payment options that incentivise productivity and quality improvement (i.e. fee per service, bundled payment); modelling of cost recovery options that balance productivity, affordability and profit<br>• ***Equipment/consumables***: dedicated operating theatre |
| Planetary Health | Healthcare is a major consumer of energy and resources and produces considerable amounts of emissions and waste. In order to protect and improve the health and wellbeing of future generations, it needs to shift towards environmentally sustainable system. | • Carbon footprint of cataract surgery<br>• Waste generated during cataract surgery | • ***Equipment/consumables***: reusable equipment, waste management<br>• ***HIS***: audit, lifecycle assessment<br>• ***Financing***: sustainable procurement |

*excluding surgical and aesthetical technical aspects, equipment and medication



For peer review only - http://bmjopen.bmj.com/site/about/guidelines.xhtml

## METHODS AND ANALYSIS

### Objectives / Scoping review questions

We aim to answer the following three questions:

1. What interventions to improve quality of cataract services have been described in the published literature?
2. Which element(s) of quality did the interventions address?
3. Where was the evidence generated (high- vs middle- vs low-income settings)?

### Protocol and registration

This protocol for this scoping review is reported according to the relevant sections of the PRISMA Extension for Scoping Reviews (PRISMA-ScR) guideline (Annex 1).[35] The protocol is registered on the Open Science Framework (https://osf.io/8gktz).

### Eligibility criteria

This scoping review will include primary research studies of any design and systematic reviews from any country that report a quality-relevant outcome for primary age-related cataract following an intervention related to quality of cataract services. We will only include studies where an intervention is compared against any alternatives (e.g. intervention vs. no intervention / current practice vs. new intervention / before vs. after implementation). Examples of relevant interventions are provided in Table 1, mapped against the eight quality elements of interest. Systematic reviews will be included only if meta-analysis is conducted for a quality-relevant outcome. If we identify systematic reviews which report narrative synthesis of quality-relevant outcomes without meta-analysis, we will review the list of included studies and include in our scoping review any that meet our eligibility criteria.

We will exclude studies assessing specific surgical techniques (e.g. phacoemulsification versus manual small incision surgery, site of anaesthesia, size of incision) and/or specific products and medications used during and around the time of surgery (e.g. monofocal versus multifocal intraocular lens, drug A versus drug B) as these are typically addressed in other systematic reviews.[3-5 21] Studies focussed exclusively on cataract services for children (aged under 18 years) will be excluded, as these services differ substantially from those for age-related cataract. We will also exclude studies reporting interventions to prevent cataract formation or progression. We will exclude studies published prior to 1990, as during the last 30 years there have been a large number of major developments in cataract services that would be expected to have changed the



For peer review only - http://bmjopen.bmj.com/site/about/guidelines.xhtml

"landscape" substantially. Service delivery models prior to this time are quite different to those currently used. There will be no language limitations. Only studies where the full text is available will be included.

**Search**

We will search MEDLINE, Embase and Global Health databases using search strategies developed by a Cochrane Eyes and Vision Information Specialist (IG). The search strategy for MEDLINE is included in Annex 2. We will examine reference lists of all included articles to identify further potentially relevant reports of studies. Field experts will be provided a list of the included studies and requested to identify further potentially relevant studies for consideration in the review.

**Selection of sources of evidence**

Covidence systematic review software will be used for screening (Veritas Health Innovation, Melbourne, Australia. Available at www.covidence.org). Each title and abstract will be screened independently by two reviewers (MY, JR, HB, AA, JB, JF, SG, WD) to exclude publications that clearly do not meet the inclusion criteria. Subsequently, the full text article will be retrieved for review if the citation seems potentially relevant and two reviewers will independently assess each article against the inclusion and exclusion criteria. Any discrepancies between the reviewers will be resolved by discussion, and a third reviewer will be consulted if necessary. A PRISMA flow diagram will be completed to summarise the study selection process.

**Data charting process**

A custom form will be developed in Excel for data charting. The form will be piloted on three studies and required amendments agreed by consensus. We anticipate a broad scope of included studies, so data charting will be an iterative process throughout the review and the data charting form will be amended as required. Each included study will be charted independently by two reviewers. Any discrepancies will be resolved by discussion, and a third reviewer will be consulted if necessary. We plan to contact study authors in the case of unclear information and will make up to three attempts by email.

**Data items**

The following data items will be collected during the data charting process:

1. Publication characteristics: title, year of publication, study design, country of origin, study setting;



For peer review only - http://bmjopen.bmj.com/site/about/guidelines.xhtml

2. Characteristics of intervention/study:
    a. Context (e.g. geographic area, target population and distribution, type of interventions (categorised by health system building block), target health practitioner, duration / frequency);
    b. Quality element(s) addressed by the intervention (as outlined in Table 1);
3. Outcome(s) of the intervention/study and whether it was reported to be effective (i.e. had an effect versus had no effect) (examples of outcomes outlined in Table 1).

**Synthesis of results**

We recognise that the indication for surgery can vary across different settings due to the prevalence of vision loss from cataract, the capacity of services and the quality and safety standards in each setting. Accordingly, we will synthesize results by World Bank country income-level (high / upper-middle / lower-middle / low)[36] and (if possible) by GBD Super-Region (High income / Latin America & Caribbean / Sub-Saharan Africa / North Africa & Middle East / Southeast Asia, East Asia & Oceania / South Asia / Central Europe, Eastern Europe & Central Asia).[37]

We will summarise findings narratively and using descriptive statistical methods as appropriate. We will map each intervention to the relevant quality element. We will visualise the findings using spider charts to show the extent of the evidence across each quality element and will plot evidence in high income countries separately to low- and middle-income countries. For each intervention, we will quantify the number of studies that were reported by the authors to be effective (versus having no effect).

**Patient and Public Involvement Statement**

This protocol was developed with input from the Commissioners of the *Lancet Global Health* Commission on Global Eye Health[38], which includes people with lived experience of vision impairment (and cataract surgery), policy makers, academics, clinicians, government eye health programme leaders and advocacy specialists.

**ETHICS AND DISSEMINATION**

Ethical approval is not required, as our review will only include published and publicly accessible information.

We will publish our findings in an open-access, peer-reviewed journal and develop an accessible summary of the results for website posting and stakeholder meetings. A summary of the results will also be included in the ongoing Lancet Commission on Global Eye Health.[38]



For peer review only - http://bmjopen.bmj.com/site/about/guidelines.xhtml

## References


1. Flaxman S, Bourne R, Resnikoff S, et al. Global Causes of Distance Vision Loss: 1990-2015 and projections to 2020. *Lancet Glob Health* 2017;5(12):e1221-e34.
2. Ramke J, Zwi AB, Lee AC, et al. Inequality in cataract blindness and services: moving beyond unidimensional analyses of social position *Br J Ophthalmol* 2017;101(4):395-400.
3. De Silva S, Riaz Y, Evans J. Phacoemulsification with posterior chamber intraocular lens versus extracapsular cataract extraction (ECCE) with posterior chamber intraocular lens for age-related cataract. *Cochrane Database Syst Rev* 2014(1)
4. De Silva S, Riaz Y, Evans J. Phacoemulsification with posterior chamber intraocular lens versus extracapsular cataract extraction (ECCE) with posterior chamber intraocular lens for age-related cataract. *Cochrane Database Syst Rev* 2014(1)
5. Riaz Y, De Silva S, Evans J. Manual small incision cataract surgery (MSICS) with posterior chamber intraocular lens versus phacoemulsification with posterior chamber intraocular lens for age-related cataract. 2013(10)
6. Kuper H, Polack S, Mathenge W, et al. Does cataract surgery alleviate poverty? Evidence from a multi-centre intervention study conducted in Kenya, the Philippines and Bangladesh. 2010;5(11):e15431.
7. Eurostat. Surgical operations and procedures statistics. Available from: https://ec.europa.eu/eurostat/statistics-explained/index.php?title=Surgical_operations_and_procedures_statistics (accessed 15 November 2019).
8. Zhao J, Xu X, Ellwein LB, et al. Cataract surgical coverage and visual acuity outcomes in rural China in 2014 and comparisons with the 2006 China Nine-Province Survey. *Am J Ophthalmol* 2018;193:62-70.
9. Silva JC, Mújica OJ, Vega E, et al. A comparative assessment of avoidable blindness and visual impairment in seven Latin American countries: prevalence, coverage, and inequality. *Rev Panam Salud Publica* 2015;37(10):13-20.
10. Yin Q, Hu A, Liang Y, et al. A two-site, population-based study of barriers to cataract surgery in rural China. *Invest Ophthalmol Vis Sci* 2009;50(3):1069-75.
11. Yorston D, Gichuhi S, Wood M, et al. Does prospective monitoring improve cataract surgery outcomes in Africa? *Br J Ophthalmol* 2002;86(5):543-47.
12. Congdon N, Suburaman G-B, Ravilla T, et al. Transforming research results into useful tools for global health: BOOST. *Lancet Glob Health* 2016;4(2):e96.
13. Donabedian A. The quality of care: how can it be assessed? *JAMA* 1988;260(12):1743-48.
14. Meuleners LB, Fraser ML, Ng J, et al. The impact of first-and second-eye cataract surgery on injurious falls that require hospitalisation: a whole-population study. *Age Ageing* 2013;43(3):341-46.
15. Ramke J, Gilbert C, Lee ACL, et al. Effective cataract surgical coverage: an indicator for measuring quality-of-care in the context of Universal Health Coverage *PLoS One* 2017;12(3):e0172342.
16. Keel S, Xie J, Foreman J, et al. Population-based assessment of visual acuity outcomes following cataract surgery in Australia: the National Eye Health Survey. *Br J Ophthalmol* 2018;102(10):1419-24.
17. Bell CM, Hatch WV, Cernat G, et al. Surgeon volumes and selected patient outcomes in cataract surgery: a population-based analysis. *Ophthalmology* 2007;114(3):405-10.
18. Singh A, Garner P, Floyd K. Cost-effectiveness of public-funded options for cataract surgery in Mysore, India. *Lancet* 2000;355(9199):180-84.
19. Joanna Briggs Institute. JBI Reviewer's manual. 2019. Available from: https://wiki.joannabriggs.org/display/MANUAL (accessed 9 March 2020).




For peer review only - http://bmjopen.bmj.com/site/about/guidelines.xhtml

20. Munn Z, Peters M, Stern C, et al. Systematic review or scoping review? Guidance for authors when choosing between a systematic or scoping review approach. *BMC Med Res Methodol* 2018;18(1):143.
21. Riaz Y, Mehta J, Wormald R, et al. Surgical interventions for age-related cataract. *Cochrane Database Syst Rev* 2006(4)
22. World Health Organization. Strategies for the prevention of blindness in national programmes: a primary health care approach (2nd ed). Geneva: World Health Organization 1997.
23. World Health Organization. What is universal coverage? 2016 Accessed 1 August 2017]. Available from: http://www.who.int/health_financing/universal_coverage_definition/en/).
24. World Health Organization. Delivering quality health services: a global imperative for universal health coverage. Geneva: WHO, 2018.
25. Institute of Medicine Committee on Health Care in America. Crossing the quality chasm: A new health system for the 21st century. Washington DC: National Academies Press 2001.
26. Health TLP. The bigger picture of planetary health. *Lancet Planet Health* 2019;3(1):e1.
27. Buchan J, Dean W, Foster A, et al. What are the priorities for improving cataract surgical outcomes in Africa? Results of a Delphi exercise. *Int Ophthalmol* 2018;38(4):1409-14.
28. Lindfield R, Vishwanath K, Ngounou F, et al. The challenges in improving outcome of cataract surgery in low and middle income countries. *Indian J Ophthalmol* 2012;60(5):464.
29. World Health Organization. Framework on integrated, people-centred health services. Geneva: WHO, 2016.
30. World Health Organization. World report on vision. Geneva: WHO, 2019.
31. Sacks E, Morrow M, Story WT, et al. Beyond the building blocks: integrating community roles into health systems frameworks to achieve health for all. *BMJ Glob Health* 2019;3(Suppl 3):e001384.
32. Day A, Donachie P, Sparrow J, et al. The Royal College of Ophthalmologists' National Ophthalmology Database study of cataract surgery: report 1, visual outcomes and complications. 2015;29(552-560)
33. Congdon N, Yan X, Lansingh V, et al. Assessment of cataract surgical outcomes in settings where follow-up is poor: PRECOG, a multicentre observational study. *Lancet Glob Health* 2013;1(1):e37-e45.
34. Ackuaku-Dogbe E, Yawson A, Biritwum R. Cataract surgical uptake among older adults in Ghana. *Ghana Med J* 2015;49(2):84-89.
35. Tricco AC, Lillie E, Zarin W, et al. PRISMA extension for scoping reviews (PRISMA-ScR): checklist and explanation. *Ann Intern Med* 2018;169(7):467-73.
36. World Bank. Country and lending groups. Available from: https://datahelpdesk.worldbank.org/knowledgebase/articles/906519-world-bank-country-and-lending-groups (accessed 2 November 2019).
37. Institute for Health Metrics and Evaluation. Frequently asked questions - what countries are in each regions? Available from: https://datahelpdesk.worldbank.org/knowledgebase/articles/906519-world-bank-country-and-lending-groups (accessed 15 November 2019).
38. Burton MJ, Faal HB, Ramke J, et al. Announcing The Lancet Global Health Commission on Global Eye Health. *Lancet Glob Health* 2019;7(12):e1612-e13.




For peer review only - http://bmjopen.bmj.com/site/about/guidelines.xhtml

**Authors' Contributions:** JR and MJB conceived the idea for the review. MY and JR drafted and revised the protocol with suggestions from MJB, NC, JMF, SG, HB, AA, APM, WD and JB. IG constructed the search.

**Corresponding Author:** Miho Yoshizaki (miho.yoshizaki1@lshtm.ac.uk)

**Funding Statement:** MJB is supported by the Wellcome Trust (207472/Z/17/Z). JR is a Commonwealth Rutherford Fellow, funded by the UK government through the Commonwealth Scholarship Commission in the UK. The Lancet Global Health Commission on Global Eye Health is supported by The Queen Elizabeth Diamond Jubilee Trust, Moorfields Eye Charity [grant number GR001061], NIHR Moorfields Biomedical Research Centre, Wellcome Trust, Sightsavers, The Fred Hollows Foundation, The SEVA Foundation, British Council for the Prevention of Blindness and Christian Blind Mission.

**Acknowledgements**:

**Competing Interests:** None declared

**Number of Tables**: 1

**Number of Figures:** 1

**Data Sharing Statement:** Data generated from this review will be available upon reasonable request from Jacqueline.Ramke@lshtm.ac.uk

**Keywords:** cataract, cataract services, quality, effectiveness, safety, person-centredness, timeliness, equity,

**Word Count: 2,052**

**Figure legend:**

Figure 1. Elements of health care quality considered in this review (modified Figure 3.2 from 'Delivering quality health services: a global imperative for universal health coverage'[24] by adding *Planetary Health*)



For peer review only - http://bmjopen.bmj.com/site/about/guidelines.xhtml

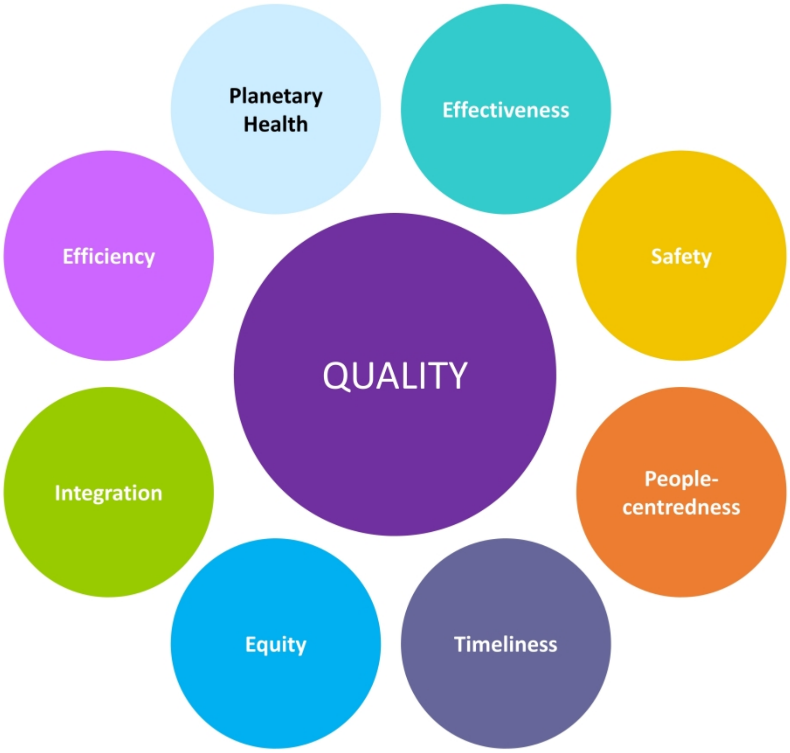


Figure 1. Elements of health care quality considered in this review (modified Figure 3.2 from 'Delivering quality health services: a global imperative for universal health coverage'24 by adding Planetary Health)

254x190mm (600 x 600 DPI)

## Annex 1: Preferred Reporting Items for Systematic reviews and Meta-Analyses extension for Scoping Reviews (PRISMA-ScR) Checklist

| SECTION | ITEM | PRISMA-ScR CHECKLIST ITEM | REPORTED ON PAGE # |
|---|---|---|---|
| **TITLE** | | | |
| Title | 1 | Identify the report as a scoping review. | 1 |
| **ABSTRACT** | | | |
| Structured summary | 2 | Provide a structured summary that includes (as applicable): background, objectives, eligibility criteria, sources of evidence, charting methods, results, and conclusions that relate to the review questions and objectives. | 2-3 |
| **INTRODUCTION** | | | |
| Rationale | 3 | Describe the rationale for the review in the context of what is already known. Explain why the review questions/objectives lend themselves to a scoping review approach. | 4-5 |
| Objectives | 4 | Provide an explicit statement of the questions and objectives being addressed with reference to their key elements (e.g., population or participants, concepts, and context) or other relevant key elements used to conceptualize the review questions and/or objectives. | 5-8 |
| **METHODS** | | | |
| Protocol and registration | 5 | Indicate whether a review protocol exists; state if and where it can be accessed (e.g., a Web address); and if available, provide registration information, including the registration number. | 9 |
| Eligibility criteria | 6 | Specify characteristics of the sources of evidence used as eligibility criteria (e.g., years considered, language, and publication status), and provide a rationale. | 9-10 |
| Information sources | 7 | Describe all information sources in the search (e.g., databases with dates of coverage and contact with authors to identify additional sources), as well as the date the most recent search was executed. | 10 |
| Search | 8 | Present the full electronic search strategy for at least 1 database, including any limits used, such that it could be repeated. | 10 |
| Selection of sources of evidence | 9 | State the process for selecting sources of evidence (i.e., screening and eligibility) included in the scoping review. | 10 |
| Data charting process | 10 | Describe the methods of charting data from the included sources of evidence (e.g., calibrated forms or forms that have been tested by the team before their use, and whether data charting was done independently or in duplicate) and any processes for obtaining and confirming data from investigators. | 10 |
| Data items | 11 | List and define all variables for which data were sought and any assumptions and simplifications made. | 10-11 |
| Critical appraisal of individual sources of evidence§ | 12 | If done, provide a rationale for conducting a critical appraisal of included sources of evidence; describe the methods used and how this information was used in any data synthesis (if appropriate). | N/A |
| Synthesis of results | 13 | Describe the methods of handling and summarizing the data that were charted. | 11 |

JBI = Joanna Briggs Institute; PRISMA-ScR = Preferred Reporting Items for Systematic reviews and Meta-Analyses extension for Scoping Reviews.

*From:* Tricco AC, Lillie E, Zarin W, O'Brien KK, Colquhoun H, Levac D, et al. PRISMA Extension for Scoping Reviews (PRISMA-ScR): Checklist and Explanation. Ann Intern Med. ;169:467–473. doi: 10.7326/M18-0850

For peer review only

**Annex 2: Example search - MEDLINE**

1. exp cataract/
2. Cataract Extraction/
3. cataract$.tw.
4. or/1-3
5. "Quality of Health Care"/
6. Quality Improvement/
7. Delivery of Health Care/
8. National Health Programs/
9. State Medicine/
10. Regional Health Planning/
11. Health Planning/
12. Health Plan Implementation/
13. Health Planning Guidelines/
14. Health Care Reform/
15. Health Resources/
16. Health Priorities/
17. Health Services Research/
18. "health services needs and demand"/
19. Needs Assessment/
20. State Health Plans/
21. Regional Health Planning/
22. Community Health Planning/
23. Hospital Planning/
24. Regional Medical Programs/
25. Health Maintenance Organizations/
26. Comprehensive Health Care/
27. Health Facility Planning/
28. Health Facility Administration/
29. Hospital Administration/
30. exp Hospitals, public/
31. exp Hospitals, private/
32. health system$.tw.

For peer review only

33. Models, Organizational/
34. Decision Making, Organizational/
35. Resource Allocation/
36. Efficiency, Organizational/
37. Organizational Innovation/
38. Delivery of Health Care, Integrated/
39. Interdisciplinary Communication/
40. Public Health/
41. Health Promotion/
42. Policy Making/
43. Program Development/
44. Program Evaluation/
45. Quality Control/
46. Quality Assurance, Health Care/
47. Benchmarking/
48. Capacity Building/
49. Health Services Accessibility/
50. Health Policy/
51. Surgical Procedures, Operative/
52. exp Surgical Equipment/
53. Health Care Rationing/
54. Medically Underserved Area/
55. Healthcare Disparities/
56. Health Status Disparities/
57. exp Attitude to Health/
58. "Patient Acceptance of Health Care"/
59. Health Education/
60. Public Opinion/
61. Health Behavior/
62. Social Behavior/
63. Superstitions/
64. exp Communication/
65. exp Culture/
66. Sex Factors/

For peer review only

67. Women's Rights/
68. Prejudice/
69. Vulnerable Populations/
70. Social Responsibility/
71. Social Welfare/
72. Urban Health Services/
73. Rural Health Services/
74. Rural Population/
75. (health adj20 (barrier$ or belie$ or inform$ or aware$ or knowledge or perceive$ or consequence$ or uptake or seek$ or underutili$ or fear$ or stigma$ or inequaliti$ or gender or logistic$ or distance$)).tw.
76. Patient Escort Service/
77. transport$.tw.
78. gender inequality.tw.
79. Mass Screening/
80. (referral adj3 (pathway$ or service$ or improve$)).tw.
81. (health worker$ or case finder$).tw.
82. or/5-81
83. Health Manpower/
84. Health Personnel/
85. Medical Staff, Hospital/
86. Nursing Staff, Hospital/
87. Personnel, Hospital/
88. Professional Competence/
89. Clinical Competence/
90. Medical Errors/
91. Professional Autonomy/
92. Leadership/
93. (leadership or motivat$).tw.
94. Motivation/
95. Organizational Innovation/
96. Personnel Selection/
97. Personnel Management/
98. Personnel Loyalty/

For peer review only

99. Job Satisfaction/
100. Staff Development/
101. "Attitude of Health Personnel"/
102. Personnel Turnover/
103. or/83-102
104. Clinical Governance/
105. Government Regulation/
106. Public Policy/
107. Public Health Practice/
108. Public Health Administration/
109. Health Plan Implementation/
110. Public-Private Sector Partnerships/
111. governance.tw.
112. or/104-111
113. (health management information system$ or HMIS).tw.
114. Management Information Systems/
115. Database Management Systems/
116. Computer Systems/
117. Point-of-Care Systems/
118. Hospital Information Systems/
119. Geographic Information Systems/
120. exp Medical Records Systems, Computerized/
121. Health Care Surveys/
122. Data Collection/
123. Data Interpretation, Statistical/
124. "Information Storage and Retrieval"/
125. Computer Literacy/
126. User-Computer Interface/
127. Attitude to Computers/
128. or/113-127
129. Delivery of Health Care, Integrated/
130. service delivery.tw.
131. decision making.tw.
132. (consensus adj3 (process$ or discuss)).tw.

For peer review only

133. stakeholder$.tw.
134. Quality Control/
135. Total Quality Management/
136. Quality Indicators, Health Care/
137. Quality Assurance, Health Care/
138. quality assurance.tw.
139. (quality adj2 improv$).tw.
140. total quality.tw.
141. continuous quality.tw.
142. quality management.tw.
143. (organisation$ adj3 cultur$).tw.
144. Disease Management/
145. Program Evaluation/
146. ((provider$ or program$) adj3 (monitor$ or evaluate$ or modif$ or practice)).tw.
147. (implement$ adj3 (improve$ or change$ or effort$ or issue$ or impede$ or glossary or tool$ or innovation$ or outcome$ or driv$ or examin$ or reexamin$ or scale$ or strateg$ or advis$ or expert$)).tw.
148. (needs adj3 assess$).tw.
149. ((education$ or learn$) adj5 (continu$ or material$ or meeting or collaborat$)).tw.
150. exp Medical audit/
151. (audit or feedback or compliance or adherence or training or innovation).ti.
152. (guideline$ adj3 (clinical or practice or implement$ or promot$)).tw.
153. exp Health Services Accessibility/
154. (outreach adj2 (service$ or visit$)).tw.
155. (intervention$ adj3 (no or usual or routine or target$ or tailor$ or mediat$)).tw.
156. usual care.tw.
157. exp Reminder Systems/
158. remind$.tw.
159. (improve$ adj3 (attend$ or visit$ or intervention$ or adhere$)).tw.
160. (increas$ adj3 (attend$ or visit$ or intervention$ or adhere$)).tw.
161. (appointment$ adj3 (miss$ or fail$ or remind$ or follow up)).tw.
162. Telephone/
163. telephone.tw.
164. Cell Phones/

For peer review only

165. Mobile Applications/
166. Remote Consultation/
167. (m-health or e-health or g-health or u-health).tw.
168. (phone$ adj1 (smart or cell)).tw.
169. (smartphone$ or cellphone$).tw.
170. (hand adj1 held device$).tw.
171. (mobile adj2 (health or healthcare or phone$ or device$ or monitor$ or comput$ or app or apps or application)).tw.
172. Internet/
173. Social Networking/
174. (email$ or text$ or message$).tw.
175. (letter or mail or mailed or print$ or brochure$ or newsletter$).tw.
176. Primary Health Care/
177. General Practitioners/ or Physicians, Family/ or Physicians, Primary Care/
178. Primary Prevention/
179. Preventive Health Services/
180. Community Health Services/
181. Community Health Nursing/
182. Health Services, Indigenous/
183. Rural Health Services/
184. Mobile Health Units/
185. (Ophthalmologist$ or Optometrist$ or Optician$ or Orthopist$ or Refractionists).tw.
186. ((Ophthalmic or eye) adj3 (surgeon$ or nurse$ or technician$ or officer$ or assistant$ or staff$)).tw.
187. Physician's Practice Patterns/
188. Professional Practice/
189. (professional adj3 (practice or develop$ or educat)).tw.
190. Education, Medical, Continuing/
191. exp nurses/
192. Specialties, Nursing/
193. Nurse's Role/
194. Education, Nursing, Continuing/
195. (nurse or nurses).tw.
196. Pharmacists/

For peer review only

197. pharmacist$.tw.
198. ((role or roles) adj3 expan$).tw.
199. (task$ adj3 shift$).tw.
200. exp Medical Records Systems, Computerized/
201. Management Information Systems/
202. Database Management Systems/
203. Computer Systems/
204. Point-of-Care Systems/
205. Hospital Information Systems/
206. ((health or healthcare) adj4 (record or management system$)).tw.
207. (decision adj5 support).ti.
208. Economics/
209. "costs and cost analysis"/
210. Cost allocation/
211. Cost-benefit analysis/
212. Cost control/
213. Cost savings/
214. Cost of illness/
215. Cost sharing/
216. "deductibles and coinsurance"/
217. Medical savings accounts/
218. Health care costs/
219. Direct service costs/
220. Drug costs/
221. Employer health costs/
222. Hospital costs/
223. Health expenditures/
224. Capital expenditures/
225. Value of life/
226. exp economics, hospital/
227. exp economics, medical/
228. Economics, nursing/
229. Economics, pharmaceutical/
230. exp "fees and charges"/

For peer review only

231. exp budgets/

232. (low adj cost).mp.

233. (high adj cost).mp.

234. (health?care adj cost$).mp.

235. (fiscal or funding or financial or finance).tw.

236. (cost adj estimate$).mp.

237. (cost adj variable).mp.

238. (unit adj cost$).mp.

239. (economic$ or pharmacoeconomic$ or price$ or pricing).tw.

240. Uncompensated Care/

241. Reimbursement Mechanisms/

242. Reimbursement, Incentive/

243. (insurance adj3 (health$ or scheme$)).tw.

244. (financial or economic or pay or payment or copayment or paid or fee or fees or monetary or money or cash or incentiv$ or disincentiv$).tw.

245. ((pay or paying or paid or cost$ or free or wait$ or qualit$) adj3 surg$).tw.

246. (will$ adj3 pay$).tw.

247. (waiting adj2 time).tw.

248. ((surgery or surgical) adj2 (experience or supervis$ or rate or rates or output or volume or uptake)).tw.

249. productivity.tw.

250. (patient adj3 (knowledge or satisfi$ or attitude$)).tw.

251. (percept$ adj3 quality).tw.

252. (follow up adj3 (appointment$ or poor or compliant or compliance)).tw.

253. exp Patient Acceptance of health Care/

254. exp Attitude to Health/

255. exp Health Behavior/

256. (barrier$ or obstacle$ or facilitat$ or enable$).tw.

257. (uptake or takeup or attend$ or accept$ or adhere$ or attitude$ or participat$ or facilitat$ or utilisat$ or utilizat$).tw.

258. (complie$ or comply or compliance$ or noncompliance$ or non compliance$).tw.

259. (encourag$ or discourage$ or reluctan$ or nonrespon$ or non respon$ or refuse$).tw.

260. (non-attend$ or non attend$ or dropout or drop out or apath$).tw.

261. Health Education/

For peer review only

262. exp Patient Education as Topic/
263. exp Health Promotion/
264. exp Counseling/
265. "Attitude of Health Personnel"/
266. (health adj2 (promotion$ or knowledge or belief$)).tw.
267. (educat$ adj2 (intervention$ or information or material or leaflet)).tw.
268. Socioeconomic Factors/
269. exp Poverty/
270. Social Class/
271. Educational Status/
272. ((school or education$) adj3 (status or level$ or attain$ or achieve$)).tw.
273. Employment/
274. Healthcare Disparities/
275. Health Status Disparities/
276. exp Medically Underserved Area/
277. Rural Population/
278. Urban Population/
279. exp Ethnic Groups/
280. Minority Groups/
281. Vulnerable Populations/
282. ((health$ or social$ or racial$ or ethnic$) adj5 (inequalit$ or inequit$ or disparit$ or equit$ or disadvantage$ or depriv$)).tw.
283. (disadvant$ or marginali$ or underserved or under served or impoverish$ or minorit$ or racial$ or ethnic$).tw.
284. (day adj3 (care or case$ or surger$)).tw.
285. (first eye adj1 cataract$).tw.
286. (second eye adj1 cataract$).tw.
287. (fellow eye adj1 cataract$).tw.
288. (simultaneous adj2 (phaco$ or phako$ or cataract$)).tw.
289. (bilateral adj2 (cataract$ surg$ or cataract$ extract$ or cataract$ remov$)).tw.
290. (sequential adj2 (cataract$ surg$ or cataract$ extract$ or cataract$ remov$)).tw.
291. Computer Simulation/
292. (virtual$ or simulat$).tw.
293. (residenc$ or resident$ or curriculum).tw.

For peer review only

294. or/129-293

295. 82 or 103 or 112 or 128 or 294

296. 4 and 295

297. epidemiologic studies/ or case-control studies/ or cohort studies/ or follow-up studies/ or longitudinal studies/ or prospective studies/ or controlled before-after studies/ or cross-sectional studies/ or historically controlled study/ or interrupted time series analysis/

298. epidemiologic methods/ or focus groups/ or interviews as topic/ or exp "surveys and questionnaires"/

299. epidemiologic research design/ or control groups/ or cross-over studies/ or double-blind method/ or meta-analysis as topic/ or network meta-analysis/ or random allocation/ or single-blind method/

300. epidemiologic methods/ or clinical trials as topic/ or feasibility studies/ or multicenter studies as topic/ or pilot projects/ or sampling studies/ or twin studies as topic/

301. randomized controlled trial/ or controlled clinical trials as topic/ or randomized controlled trials as topic/

302. comparative study/ or evaluation studies/ or meta-analysis/ or multicenter study/ or "systematic review"/ or validation studies/

303. Educational Measurement/

304. "Outcome and Process Assessment (Health Care)"/ or "Outcome Assessment (Health Care)"/

305. (cross adj1 section$).tw.

306. (cohort or intervention or prospective or comparative).tw.

307. (questionnaire$ or survey$).tw.

308. focus group$.tw.

309. (randomized or randomised or randomly).tw.

310. or/297-309

311. 296 and 310

312. (glaucoma$ or trabeculectom$ or angle closure or diabetic retinopath$ or keratoplast$ or keratopath$ or pseudoexfoliat$ or macula$ edema or macula$ oedema or retinal detachment$ or macula$ degeneration or scleral buckl$ or dry eye$ or uveitis or endothelial or endothelium or myopia or myopic or exotropia or amblyopia).ti.

313. (IOL$ or intraocular lens$ or trifocal or bifocal or multifocal or monofocal).ti.

314. (phacoemulsificat$ or capsulorhexis or wavefront or lensectomy or femtosecond or ECCE or SICS or MSICS or small incision or suture).ti.

For peer review only

315. (incidence or incident or prevalence).ti.

316. (dexamethasone or povidine or iodine or diclofenac or prednisolone or indomethacin or betaxolol or triamcinolone or nepafenac or corticosteroid$ or fluorouracil or bevacizumab or ranibizumab or radiation or ultrasound or intracameral or intravitreal or pseudophak$ or limbal or PMMA).ti.

317. optical coherence tomography.ti.

318. (genotyp$ or phenotyp$ or biomarker$ or genes or chromosome$ or mutation$).ti.

319. or/312-318

320. 311 not 319

321. exp case reports/

322. (case$ adj3 (report$ or stud$ or series)).tw.

323. 321 or 322

324. 320 not 323

325. limit 324 to (comment or editorial or letter or observational study)

326. 324 not 325

327. limit 326 to yr="1990 -Current"

For peer review only
